# Identification of core genes associated with the anti-atherosclerotic effects of Salvianolic acid B and immune cell infiltration characteristics using bioinformatics analysis

**DOI:** 10.1186/s12906-022-03670-6

**Published:** 2022-07-16

**Authors:** Zheng Jin, Huanyi Zhao, Yuan Luo, Xiushen Li, Jiayan Cui, Jing Yan, Pingzhen Yang

**Affiliations:** 1 ZhuJiang Hospital of Southern Medical University, Guangzhou, 510285 Guangdong China; 2 First Affiliated Hospital of Guangzhou University of Chinese Medicine, Guangzhou, 510405 Guangdong China; 3 Guangzhou University of Chinese Medicine, Guangzhou, 510405 Guangdong China; 4 Department of Obstetrics and Gynecology, Shenzhen University General Hospital, Shenzhen, China

**Keywords:** Atherosclerosis, Immune cell infiltration, Bioinformatic analysis, Salvianolic acid B

## Abstract

**Background:**

Atherosclerosis (AS) is the greatest contributor to pathogenesis of atherosclerotic cardiovascular disease (ASCVD), which is associated with increased mortality and reduced quality of life. Early intervention to mitigate AS is key to prevention of ASCVD. *Salvianolic acid B* (Sal B) is mainly extracted from root and rhizome of *Salvia Miltiorrhiza Bunge*, and exerts anti-atherosclerotic effect. The purpose of this study was to screen for anti-AS targets of Sal B and to characterize immune cell infiltration in AS.

**Methods:**

We identified targets of Sal B using SEA (http://sea.bkslab.org/) and SIB (https://www.sib.swiss/) databases. GSE28829 and GSE43292 datasets were obtained from Gene Expression Omnibus database. We identified differentially expressed genes (DEGs) and performed enrichment analysis. Weighted gene co-expression network analysis (WGCNA) was used to determine the most relevant module associated with atherosclerotic plaque stability. Intersecting candidate genes were evaluated by generating receiver operating characteristic (ROC) curves and molecular docking. Then, immune cell types were identified using CIBERSOFT and single-sample gene set enrichment analysis (ssGSEA), the relationship between candidate genes and immune cell infiltration was evaluated. Finally, a network-based approach to explore the candidate genes relationship with microRNAs (miRNAs) and Transcription factors (TFs).

**Results:**

MMP9 and MMP12 were been selected as candidate genes from 64 Sal B-related genes, 81 DEGs and turquoise module with 220 genes. ROC curve results showed that MMP9 (AUC = 0.815, *P*<0.001) and MMP12 (AUC = 0.763, *P*<0.001) were positively associated with advanced atherosclerotic plaques. The results of immune infiltration showed that B cells naive, B cells memory, Plasma cells, T cells CD8, T cells CD4 memory resting, T cells CD4 memory activated, T cells regulatory (Tregs), T cells gamma delta, NK cells activated, Monocytes, and Macrophages M0 may be involved in development of AS, and the candidate genes MMP9 and MMP12 were associated with these immune cells to different degrees. What’ s more, miR-34a-5p and FOXC1, JUN maybe the most important miRNA and TFs.

**Conclusion:**

The anti-AS effects of Sal B may be related to MMP9 and MMP12 and associated with immune cell infiltration, which is expected to be used in the early intervention of AS.

**Supplementary Information:**

The online version contains supplementary material available at 10.1186/s12906-022-03670-6.

## Introduction

Atherosclerosis is the fundamental underlying pathology of atherosclerotic cardiovascular disease (ASCVD). Chronic accumulation of vessel-occluding plaques in the subendothelial intimal layer of large and medium arteries results in significant stenosis that restricts blood flow, and causes critical tissue hypoxia and vascular inflammation [1]. A systematic analysis of deaths from 137 countries showed that sudden rupture of unstable atherosclerotic plaques, which results in myocardial infarction (MI) and stroke, is the leading cause of ASCVD-related death worldwide [2, 3]. Genome-wide association studies have shown that the innate and adaptive immune responses contribute to atherosclerosis [4]. The innate immune response can be activated by cholesterol crystals through inflammasome activation [5], and the adaptive immune response can be activated recognition of native oligopeptides derived from ApoB100 [6]. Antigen-presenting cells can present ApoB-derived peptides, resulting in T-cell activation, pathogenic Th1 cell differentiation, or activation of anti-inflammatory regulatory T cells (Tregs) [7]. However, the mechanisms by which this antigen presentation leads to immune cell activation and differentiation, or induction, of the anti-inflammatory response has not been characterized.

*Salvianolic acid B* (Sal B), a water-soluble substance extracted from the root and rhizome of *Salvia Miltiorrhiza*, is a phenolic acid with a molecular weight of 748 Da. Studies have shown that *Salvia Miltiorrhiza* and its components prevent vascular diseases, particularly cardiovascular diseases, through induction of vascular smooth muscle cell (VSMC) proliferation and migration, inhibition of foam cell formation, and local aggregation of inflammation cells [8, 9]. Sal B has also been shown to exert cardioprotective effects against cardiac ischemic injury [10, 11], reperfusion injury [9], and heart failure induced by pressure overload [12]. Guo et al. [13] have reported that Sal B have protective effects on vascular endothelial cell via activation of the Keap1-Nrf2-ARE pathway. Recently, Sal B was shown to inhibit chemical and mechanical activation of Piezo1 channels, resulting in prevention of atherosclerotic lesion formation [14]. Although the cardiovascular effects of the components of *Salvia Miltiorrhiza* have received increased attention, the mechanisms of action of the various components are unclear.

**Fig. 1:**
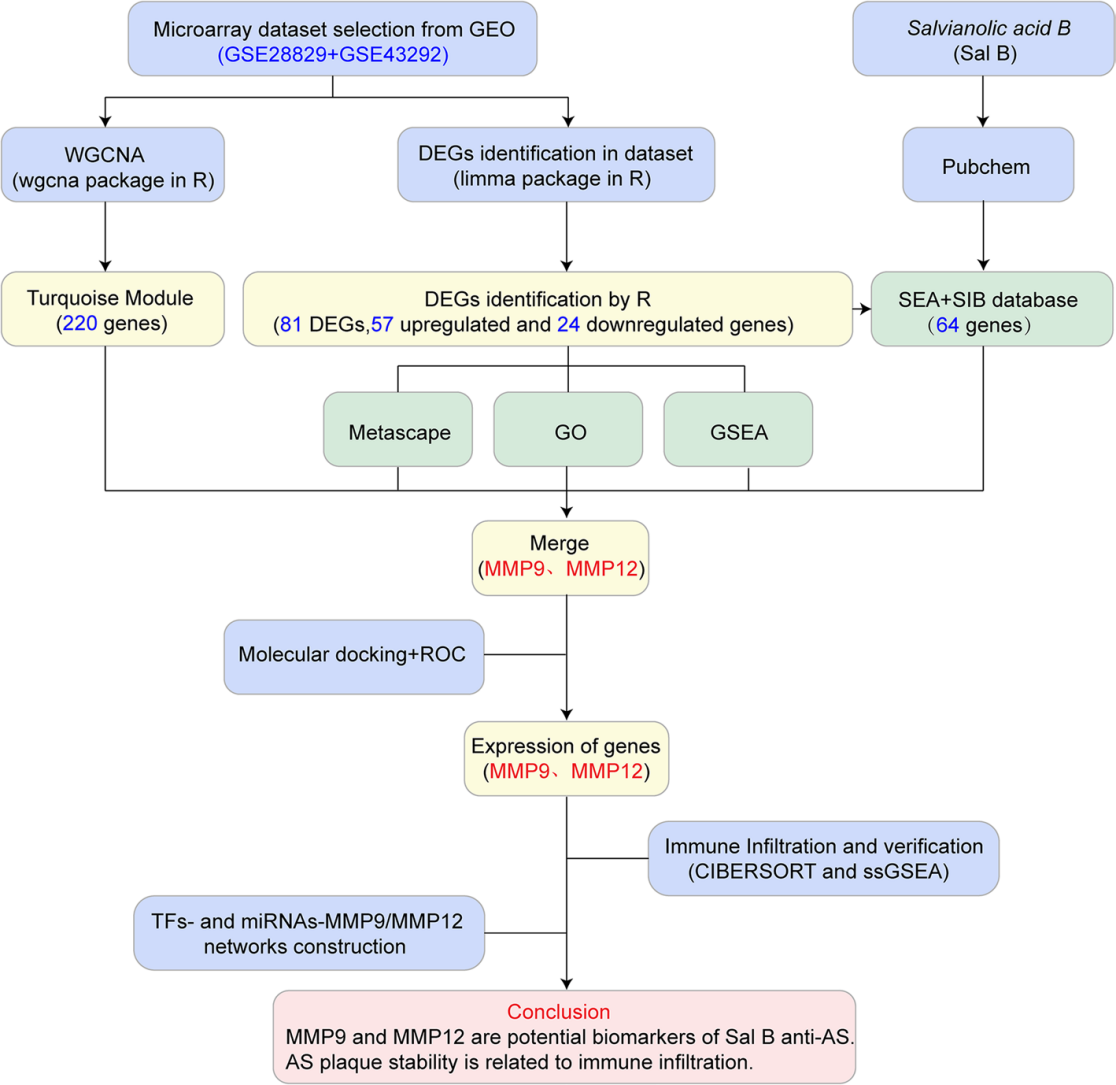
Workflow of the strategies to elucidate the mechanisms of Sal B on AS plaque stability

In this study, Sal B anti-AS candidate genes were screened using Sal B targets, DEGs, and WGCNA, and verified using ROC curves and molecular docking. The CIBERSORT and ssGSEA methods were used to study differences in immune cells between patients with early atherosclerotic plaque and patients with advanced atherosclerotic plaque. In addition, the correlation between candidate genes and immune cells was analyzed to further explore the immune mechanisms of onset and development of AS. What’ s more, both TFs-candidate genes and miRNAs-candidate genes relationship networks were explored by NetworkAnalyst platform. The workflow of the study is shown in Fig. 1.

## Methods and materials

### Data collection

The 2D structure of Sal B was obtained by searching the PubChem compound database (https://pubchem.ncbi.nlm.nih.gov/), and uploaded onto the SEA database (http://sea.bkslab.org/) [15] and the SIB database (https://www.sib.swiss/) [16]. Target determination was limited to “*Homo sapiens*” to predict the targets of Sal B in humans. The identified targets are summarized Supplementary Table 1.

Clinical datasets were obtained from the Gene Expression Omnibus (GEO) database (https://www.ncbi.nlm.nih.gov/geo/) using the following keywords: (Atherosclerosis) AND “*Homo sapiens*” (porgn:__txid9606) AND “Expression profiling by array” AND “Series.” The screening standards included the following: microarray datasets for gene expressions in whole blood; microarray datasets from patients with early atherosclerotic plaques and advanced atherosclerotic plaques; no drug treatment. The GSE28829 [17] and GSE43292 [18] datasets were screened for in-depth analysis.

### Differential expression analysis and functional analysis

We used the “limma” package to identify DEGs in the GSE28829 and GSE43292 datasets. A volcano plot and heatmap were generated to show DEGs. DEGs with adjusted *P* < 0.05 and |log2 FC| > 1 were considered statistically significant (Supplementary Table 2). Thereafter, we defined the species as “*Homo sapiens*” and used the STRING 11.0 database (https://string-db.org/) to construct the protein-protein interaction (PPI) network of Sal B anti-AS and downloaded the file in TSV format. With the software Cytoscape, the PPI network was visualized and performed topology analysis (Supplementary Table 3). Pathway and process enrichment analysis via Metascape (http://metascape.org/gp/index.html). To explore the functions of genes, we performed Gene Ontology (GO) (Supplementary Table 4) analysis and Gene set enrichment analysis (GSEA) (Supplementary Table 5) with the use of the “clusterProfiler” package [19]. *P* < 0.05 was considered statistically significant.

### Weighted gene co-expression network analysis (WGCNA)

We used WGCNA to construct an mRNA co-expression network based on the GSE28829 and GSE43292 datasets. DEGs were clustered into modules according to scale-free topology, and were labeled with different colors using the average linkage hierarchical clustering method. The module with the highest correlation coefficient and gene significance was selected for further analyses (Supplementary Table 6). We used the R package “WGCNA” to perform WGCNA analysis [20].

### Candidate gene screening and evaluation

This study evaluated common genes among Sal B targets, DEGs, and targets identified using WGCNA. We generated receiver operating characteristic (ROC) curves using the R package “pROC” to determine efficacy, and the area under the curve (AUC) was calculated to determine the predictive effects of the algorithms [21]. A two-sided *P* < 0.05 indicated statistical significance. Differential expression of candidate genes analysis used the R package “ggpubr.” *P* < 0.05 was considered statistically significant.

### Molecular docking

The Sal B mol2 file was downloaded from the PubChem database. Then, target proteins were downloaded from the PDB database and imported into Discovery Studio. The model was pretreated by dehydrating, hydrogenation and etc., to prepare the target library and the Dock Ligands module was used to dock the target receptor and ligands from the small molecule library for Libdock molecular docking. PyMOL and AutoDockTools [22] were used to dehydrate, delete the original ligand, and hydrogenate the proteins, and the proteins were saved in PDBQT format as the docking receptor. AutoDock Vina software was used to calculate the semiflexible molecular docking between Sal B and candidate genes, which estimated the favorability of binding of small molecules to proteins mainly through binding free energy.

### Analysis of immune infiltration

The CIBERSORT algorithm [23] was used to filter 22 kinds of the immune cells. *P* < 0.05 was considered statistically significant. We used the “corrplot” package to show the relative percentages of the different types of immune cells and generated correlation heatmap to visualize the proportions of infiltrating immune cells between patients with early atherosclerotic plaque and patients with advanced atherosclerotic plaques, and to evaluate correlations among the 22 types of infiltrating immune cells. The “reshape2,” “ggpubr,” “ggExtra” packages were used to analyze the differences in distributions of immune cells between patients with early atherosclerotic plaques and patients with advanced atherosclerotic plaques. The single-sample gene set enrichment analysis (ssGSEA) was used to verification immune cells infiltration, which by using “GSVA” package in the R software. Then, We evaluated the relationship between candidate genes and immune cells.

### Prediction of TFs and miRNAs engage with candidate genes

Transcription factors (TFs) are the protein that attaches to a particular gene and governs the rate of transcription of genetic information [24]. We have utilized the NetworkAnalyst [25] platform to locate topologically credible TFs from the JASPAR [26] database that bind to our candidate genes. What’ s more, prediction of miRNAs engage with candidate genes were identified using the Tarbase [27]. Both TFs-diagnostic genes and miRNAs-diagnostic genes interaction networks were illustrated in Cytoscape. This tool helps researchers filter top miRNAs with high degrees and lead to the effective biological hypothesis.

## Results

### Sal B-related targets and DEGs in GSE28829 and GSE43292 datasets

**Fig. 2:**
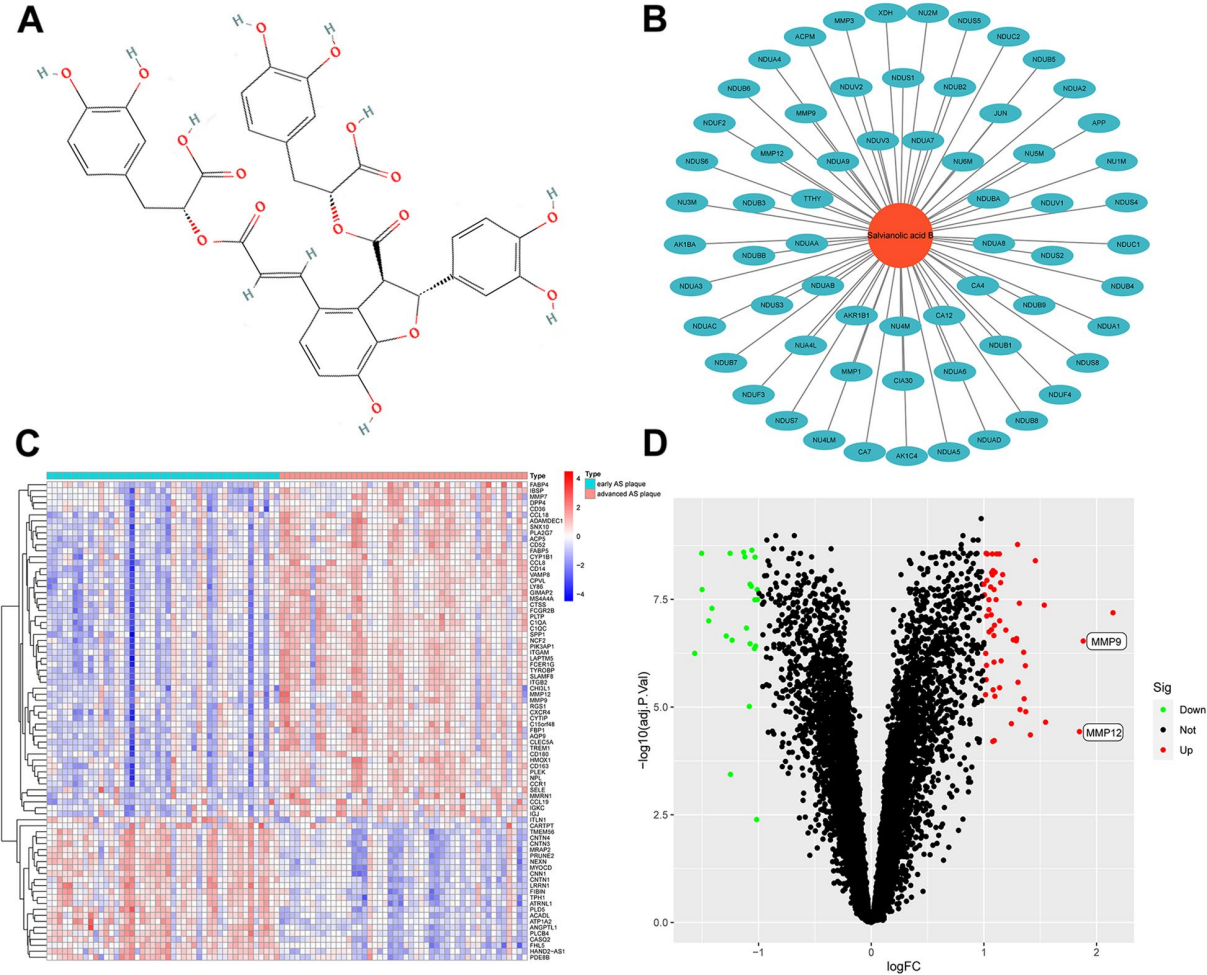
Sal B-related targets and DEGs between patients with early atherosclerotic plaques (early AS plaque) and patients with advanced atherosclerotic plaques (advanced AS plaque) in the GSE28829 and GSE43292 databases. **A** Structure of Sal B. **B** Sal B target genes was generated by using Cytoscape software. **C** Heatmap was generated by using R software for 81 DEGs. **D** Volcano plot was generated by using R software

The PubChem (https://pubchem.ncbi.nlm.nih.gov) database was used to retrieve the 2D chemical structure of Sal B (Fig. 2A). The image of the Sal B 2D chemical structure was uploaded on the similarity ensemble approach (SEA) and Swiss Institute of Bioinformatics (SIB) (probability of gene compound interaction is 0.1) databases to determine predicted targets of Sal B. Each of the predicted targets was inputted into the UniProt database for screening. We only screened for human targets that have been annotated and reviewed. Potential targets of Sal B were identified by eliminating duplicate and nonstandard targets, which resulted in identification of 64 candidate genes (Fig. 2B).

GSE28829 and GSE43292 datasets were downloaded from GEO, which were merged, filtered and normalized by R. 81 DEGs were identified between patients with early atherosclerotic plaques and patients with advanced atherosclerotic plaques, and a heat map was generated to show the expression of the 81 DEGs between the two groups (Fig. 2C). As shown in the volcano plot in Fig. 2D, red indicates up-regulated genes (57), green indicates down-regulated genes (24), and black indicates no difference in gene expression between patients with early atherosclerotic plaques and patients with advanced atherosclerotic plaques.

### Enrichment analyses

**Fig. 3:**
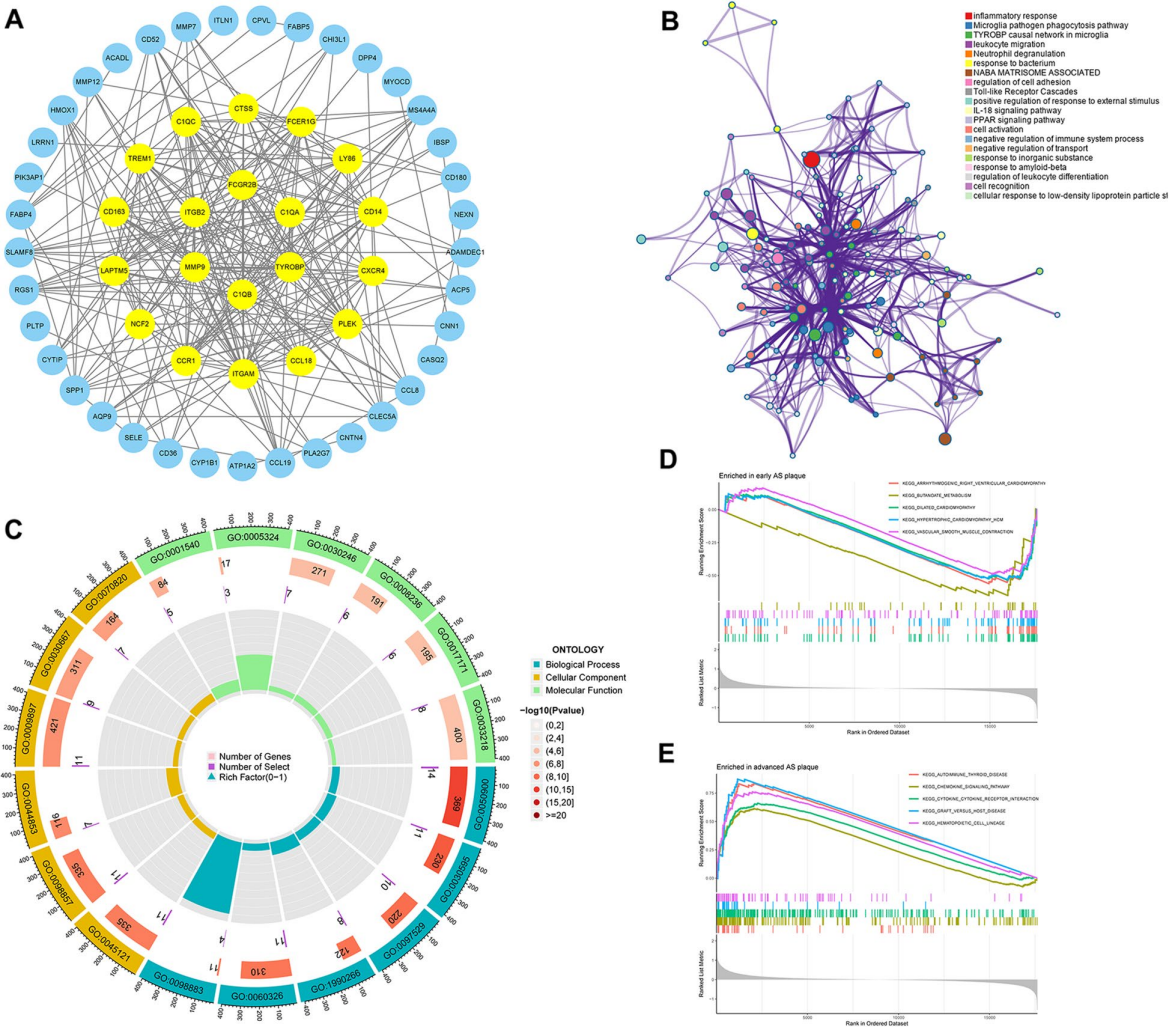
PPI network construction and enrichment analyses. **A** PPI network construction and topology analysis by Cytoscape. The yellow nodes are core genes obtained by topology analysis. **B** Pathway and process enrichment analysis network colored by cluster ID, where nodes that share the same cluster ID are typically close to each other via Metascape. **C** GO circlize was generated by using R software. **D** GSEA of patients with early atherosclerotic plaques for top 5 terms by using R software. **E** GSEA of patients with advanced atherosclerotic plaques was generated for top 5 terms by using R software

The DEGs in GSE28829 and GSE43292 were imported into the STRING database to obtain their interaction relationship, and a scoring value > 0.4 was selected as the high confidence basis for protein interactions. Next, The interaction network data were imported into Cytoscape, and the results showed a total of 57 nodes and 278 edges. Then, we performed topology analysis, 20 targets were further screened as the core genes (Fig. 3A). What’ s more, we introduced the DEGs into Metascape, terms with a *p*-value < 0.01, a minimum count of 3, and an enrichment factor > 1.5 are collected and grouped into clusters based on their membership similarities. We found that AS plaque stability were related to inflammatory response, Toll-like receptor cascades, negative regulation of immune system process,etc. (Fig. 3B). The results of GO enrichment analysis showed that changes in stability of AS were associated with neutrophil activation involved in immune response, regulation of immune effector process, immune response-regulating signaling pathway, regulation of cytokine production involved in immune response, regulation of inflammatory response, etc. (Fig. 3C). GSEA results showed that, patients with early atherosclerotic plaques samples were involved in Arrhythmogenic right ventricular cardiomyopathy, Dilated cardiomyopathy, Hypertrophic cardiomyopathy, etc. patients with advanced atherosclerotic plaques samples were involved in Autoimmune thyroid, Chemokine signaling pathway, Cytokine-cytokine receptor interaction, etc. (Fig. 3D-E).

### Weighted gene co-expression network analysis

**Fig. 4:**
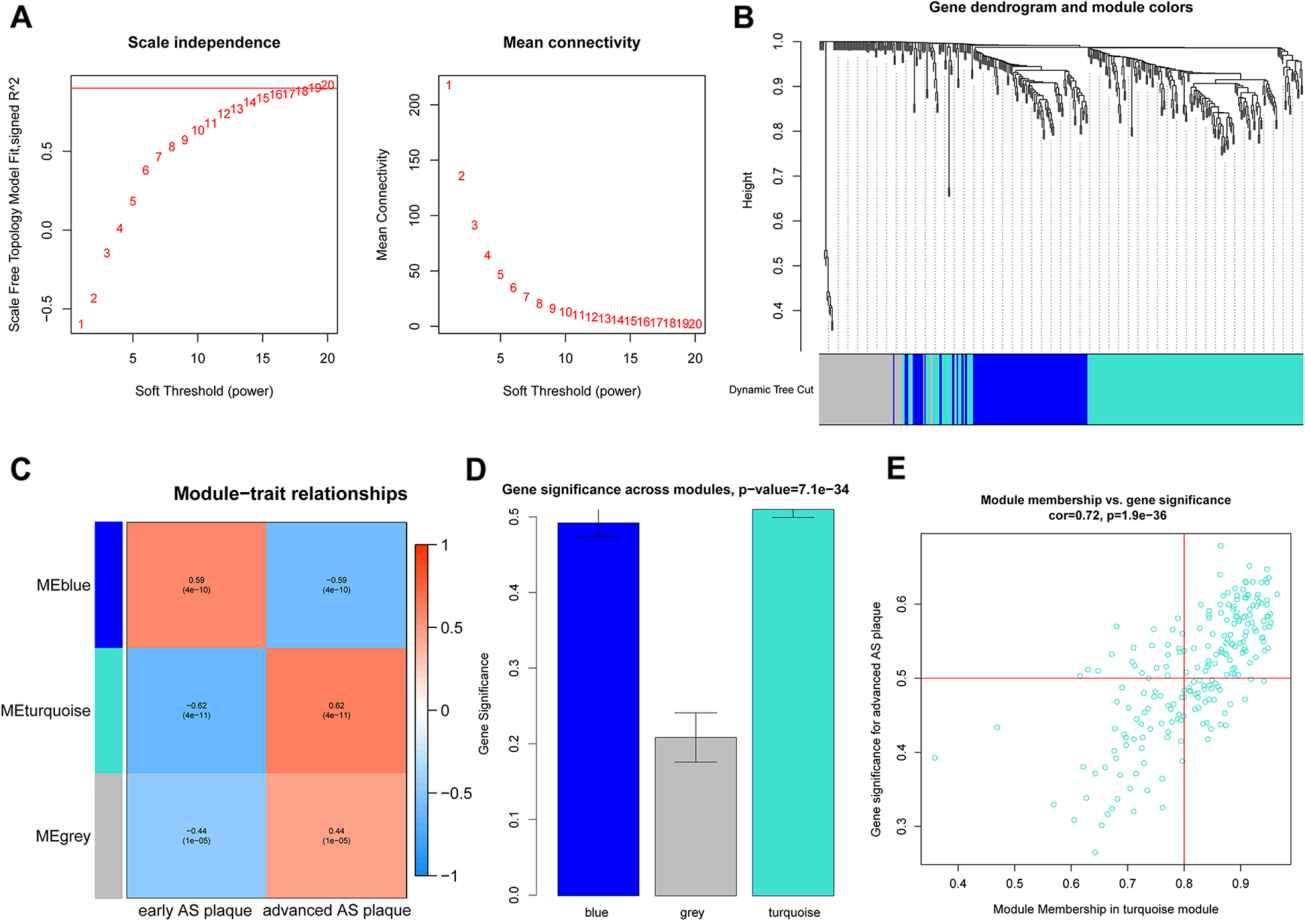
Weighted gene co-expression network analysis revealed gene co-expression networks in GSE28829 and GSE43292. **A** Analysis of the scale-free fit index and the mean connectivity for various soft-thresholding powers. **B** Clustering dendrogram of DEGs related to AS plaque stability. **C** Relationships of consensus modules with samples. Each color represents a specific gene module. **D** Gene significance across modules. **E** Gene significance for patients with advanced atherosclerotic plaques is represented in the turquoise module

For WGCNA, 0.9 was used as the correlation coefficient threshold, and the soft-thresholding power was set to 20 (Fig. 4A). Weighted gene co-expression network analysis was performed using the average linkage hierarchical clustering method, 3 modules were related to AS plaque stability with different colors were built (Fig. 4B-C). The turquoise module was the highest correlation coefficient and gene significance, with 220 genes (Fig. 4D-E).

### Candidate genes screening and verification

**Fig. 5:**
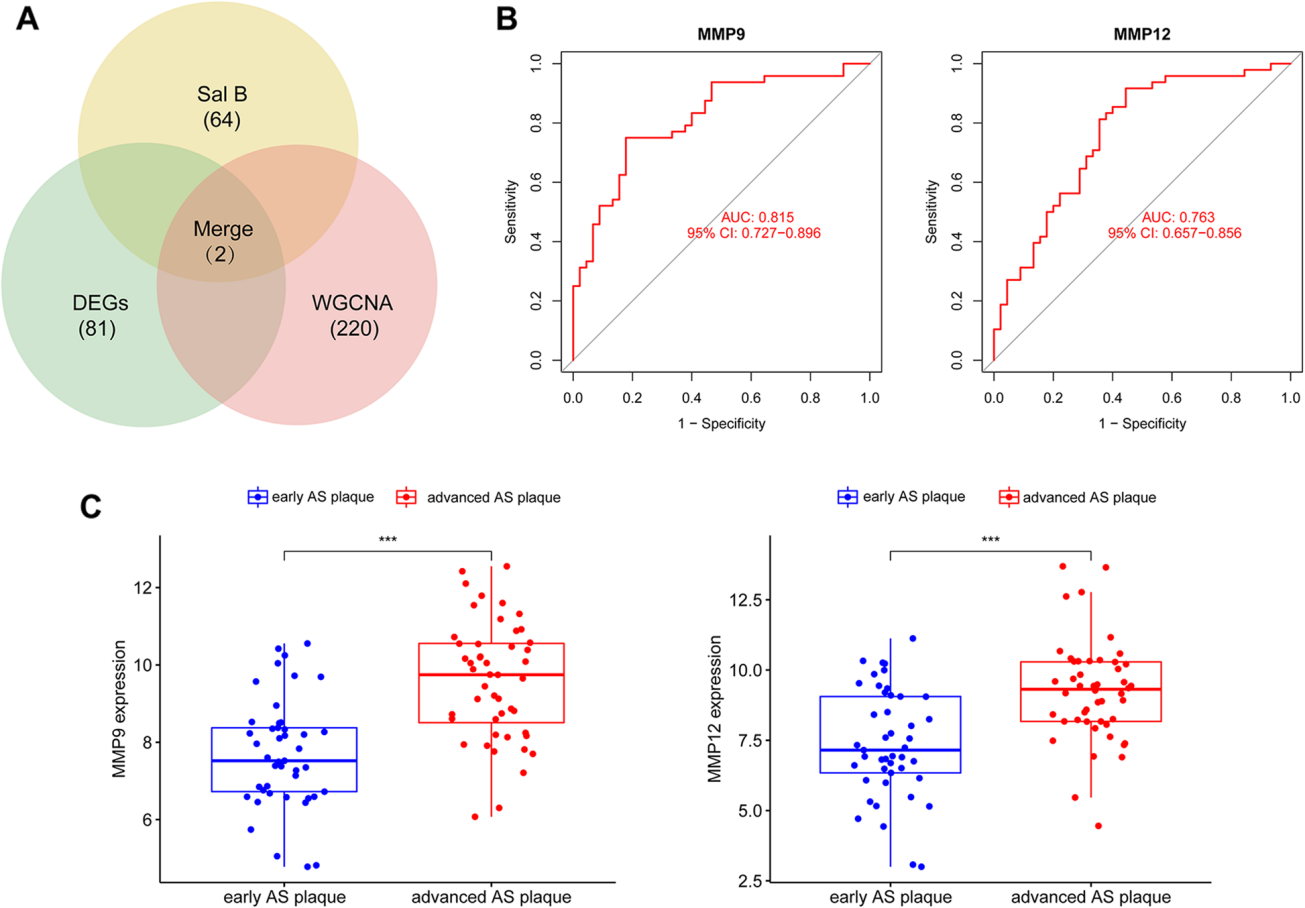
Candidate genes screening and verification of Sal B anti-AS targets. **A** The Venn diagram showing the intersection of Sal B targets, DEGs and turquoise module obtained genes, wherein 2 candidate genes identified. **B** The receiver operating characteristic (ROC) curve for the diagnostic value of MMP9 and MMP12. **C** MMP9 and MMP12 were significantly upregulated in patients with advanced atherosclerotic plaques samples

Screening for intersecting genes obtained from Sal B targets (64 genes), DEGs (81 genes), and WGCNA (220 genes in the turquoise module) identified MMP9 and MMP12 as important candidate genes (Fig. 5A). ROC curves were generated for MMP9 and MMP12, and had AUCs of 0.815 and 0.763, respectively (Fig. 5B), The expression levels of MMP9 (*p*<0.001) and MMP12 (*p*<0.001) were upregulated in patients with advanced atherosclerotic plaques (Fig. 5C), which indicated that MMP9 and MMP12 were likely important genes targeted by Sal B anti-AS.

### Molecular docking

**Fig. 6:**
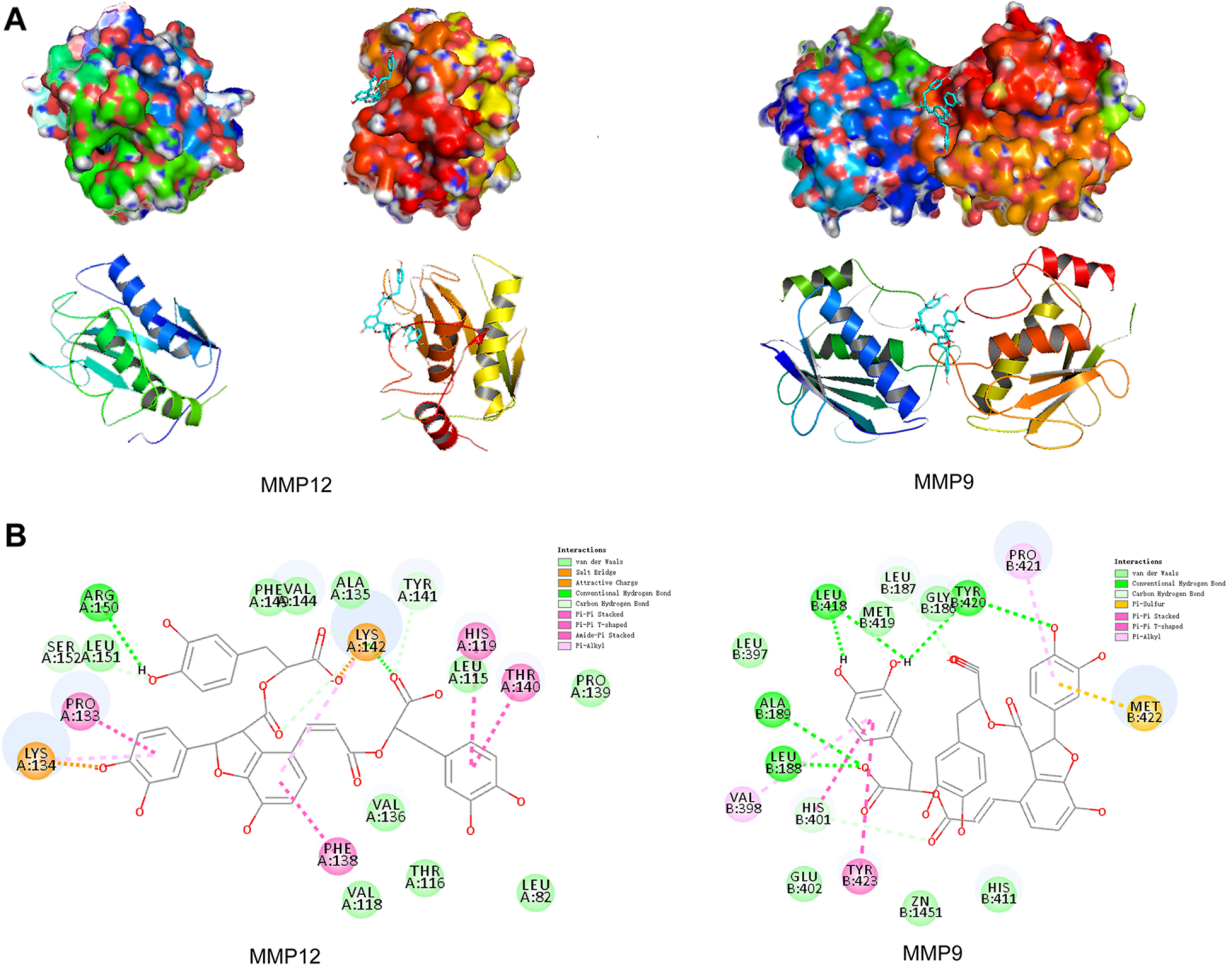
Molecular docking. **A** Docking diagram of Sal B with MMP9 and MMP12 using Pymol software. **B** Docking diagram of Sal B with MMP9 and MMP12 by Discovery Studio

The target genes selected for molecular docking analysis were MMP9 and MMP12. The binding free energy of MMP9-Sal B was − 6.8 kcal·mol^− 1^ and the binding free energy of MMP12-Sal B was − 9.2 kcal· mol^− 1^. Binding energies less than − 5.0 kcal·mol^− 1^ indicated that Sal B had favorable binding ability to both MMP9 and MMP12 [28]. The docking models were visualized using Pymol software (Fig. 6A). Discovery Studio was used to analyze the interaction modes of the combinations with the lowest binding energies. The results showed that the main interaction types were C-H bond, H-bond and Pi-Pi conjugation, which jointly maintained the binding stability of the ligand and receptor protein (Fig. 6B).

### Immune cells infiltration

**Fig. 7:**
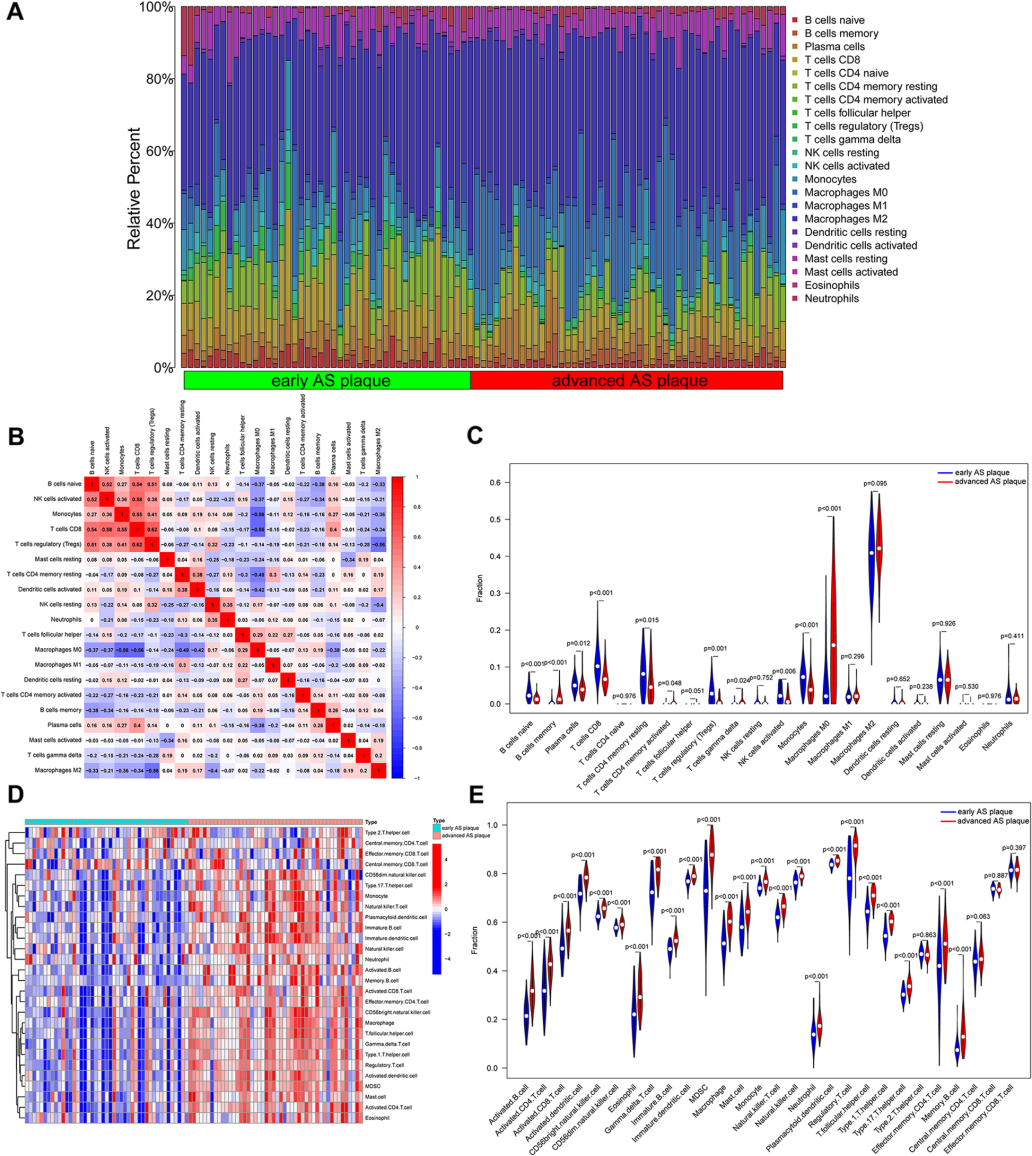
Evaluation of immune infiltration. **A** Bar charts of the proportions of 22 immune cell types in patients with early atherosclerotic plaques and advanced atherosclerotic plaques by CIBERSORT. **B** Heatmap of correlations among the 22 immune cell types by CIBERSORT. **C** Violin diagram of the proportions of 22 immune cell types between patients with early atherosclerotic plaques and advanced atherosclerotic plaques by CIBERSORT. **D** Heatmap of immune cells difference by ssGSEA. **E** Violin diagram of immune cells difference by ssGSEA

The CIBERSORT algorithm was used to analyze immune cell infiltration, and to determine the proportions of different types of immune cells in patients with advanced atherosclerotic plaques and in patients with early atherosclerotic plaques. The results showed that the two groups displayed distinct and group-biased clustering. The proportions of the 22 immune cell types are shown as a bar plot (Fig. 7A). Macrophages were the most abundant immune cells in both groups. T cells regulatory (Tregs) had the strongest positive correlation with T cells CD8 (0.62). In addition, T cells CD8 had the strongest negative correlation with Macrophages M2 (− 0.56), Macrophages M0 had the strongest negative correlation with T cells regulatory (Tregs) and T cells CD8 (− 0.56), as shown in Fig. 7B. Eleven types of immune cells were differentially present in plaques of patients with early atherosclerotic plaques and patients with advanced atherosclerotic plaques. Patients with advanced atherosclerotic plaques had higher proportions of B cells memory (*P*<0.001), Plasma cells (*P* = 0.012), T cells CD4 memory activated (*P* = 0.048), T cells gamma delta (*P* = 0.024), and Macrophages M0 (*P*<0.001), and lower proportions of B cells naive (*P*<0.001), T cells CD8 (*P*<0.001), T cells CD4 memory resting (*P* = 0.015), T cells regulatory (Tregs) (*P* = 0.001), NK cells activated (*P* = 0.006), and Monocytes (*P*<0.001) than those in patients with early atherosclerotic plaques (Fig. 7C). Single-sample gene set enrichment analysis (ssGSEA) method verified the difference of above immune cells between patients with early atherosclerotic plaques and patients with advanced atherosclerotic plaques (Fig. 7D-E).

### Immune cells correlation with candidate genes

**Fig. 8:**
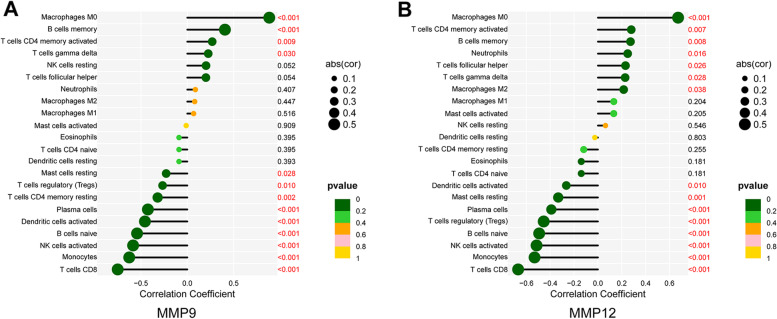
Analysis of correlation between candidate genes and immune cells. **A** Correlation between MMP9 and immune cells by using R software. **B** Correlation between MMP12 and immune cells by using R software

Correlation analysis showed that MMP9 was positively associated with M0 macrophages (*P*<0.001), B cells memory (*P*<0.001), T cells CD4 memory activated (*P* = 0.009), T cells gamma delta (*P* = 0.030), and negatively associated with Mast cells resting (*P* = 0.028), T cells regulatory (Tregs) (*P* = 0.010), T cells CD4 memory resting (*P* = 0.002), Plasma cells (*P*<0.001), Dendritic cells activated (*P*<0.001), B cells naive (*P*<0.001), NK cells activated (*P*<0.001), Monocytes (*P*<0.001), T cells CD8 (*P*<0.001) (Fig. 8A). Furthermore, MMP12 was positively associated with M0 macrophages (*P*<0.001), T cells CD4 memory activated (*P* = 0.007), B cells memory (*P* = 0.008), Neutrophils (*P* = 0.016), T cells follicular helper (*P* = 0.026), T cells gamma delta (*P* = 0.028), Macrophages M2 (*P* = 0.038), and negatively associated with Dendritic cells activated (*P* = 0.010), Mast cells resting (*P* = 0.001), Plasma cells (*P*<0.001), T cells regulatory (Tregs) (*P*<0.001), B cells naive (*P*<0.001), NK cells activated (*P*<0.001), Monocytes (*P*<0.001), T cells CD8 (*P*<0.001) (Fig. 8B).

### Candidate genes-miRNAs and candidate genes-TFs network construction

**Fig. 9:**
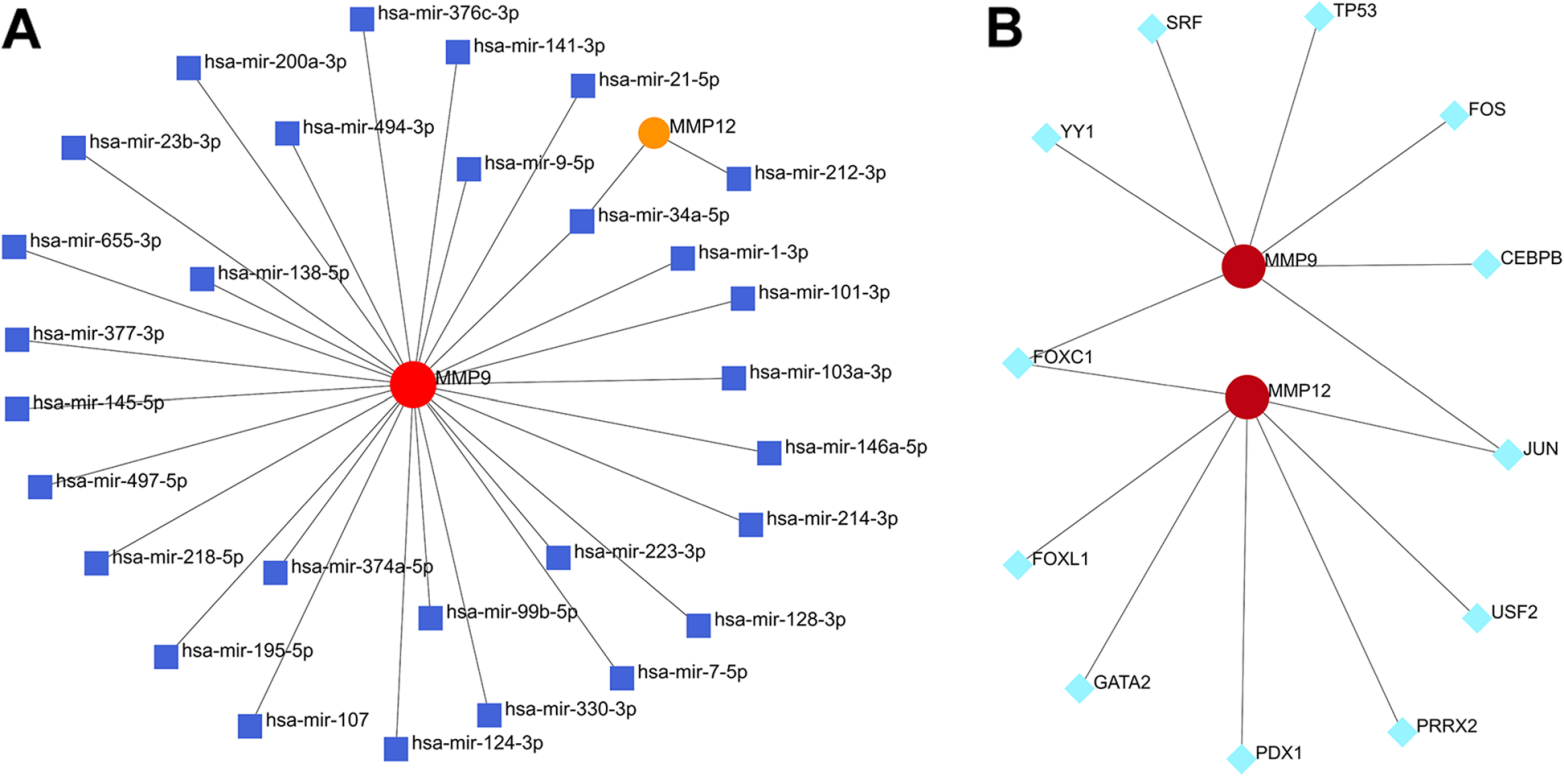
The interaction network of MMP9/MMP12-miRNAs and MMP9/MMP12-TFs. **A** The interconnected regulatory interaction network of MMP9/MMP12-miRNAs. The square nodes are miRNAs, and MMP9/MMP12 interact with miRNAs as circle nodes. **B** The cohesive regulatory interaction network of MMP9/MMP12-TFs obtained from the Network Analyst. The square nodes are TFs, and MMP9/MMP12 interact with TFs as circle nodes

To identify changes happening at the transcriptional level, we employed a network-based approach to explore the candidate genes relationship with miRNAs and TFs, candidate genes interaction with miRNAs and TFs as shown in Fig. 9A-B. It has been ascertained that 29 miRNAs and 12 TFs regulatory signatures regulate with more than one common DEGs, which essentially indicates a strong interference between them. It is worth pointing out that miR-34a-5p and FOXC1, JUN maybe the most important miRNA and TFs.

## Discussion

According to a WHO epidemiological study, heart disease has been the leading global cause of death for the last 20 years [29]. Atherosclerosis is a common pathological feature of cardiovascular disease, and conversion of AS plaques from stable to unstable can result in rupture, which is a significant cause of cardiovascular events. Although treatment of AS has improved, the incidence and mortality of ASCVD remains high [30]. *Salvianolic acid B* is derived from *Salvia Miltiorrhiza Bunge*, a plant of Labiatae. Studies have shown that Sal B can improve MI/R damage [31], inhibit cardiomyocyte hypertrophy [32], and inhibit atherosclerosis [33]. In addition, a recent study have shown that immune cell infiltration plays an important role in the onset and development of cardiovascular disease [34], and characterization of the role of infiltrating immune cells in development of AS is necessary. In this study, we used comprehensive bioinformatics analysis to show that MMP9 and MMP12 are potential biomarkers of Sal B modulation of AS plaque stability.

In our study, 64 Sal B-related targets were obtained using the SEA and SIB databases. Comparison between samples from patients with advanced atherosclerotic plaques and patients with early atherosclerotic plaques resulted in identification of 81 DEGs, including 57 up-regulated genes and 24 down-regulated genes. The enrichment of DEGs was determined using Metascape, GO analyses and GSEA. The results showed that AS plaque stability was mainly related to inflammation and immunity. 3 significant co-expression modules were constructed using cluster analysis of the genes with the same expression patterns in the patients with advanced atherosclerotic plaques and patients with early atherosclerotic plaques. The turquoise module had the highest correlation and gene significance, contained 220 genes. MMP9 and MMP12 were obtained by 64 Sal B-related genes, 81 DEGs, and 220 turquoise module genes intersection, which were verificated by using ROC curve analysis and molecular docking analysis.

*Salvianolic acid B* (Sal B) is one of the most bioactive compounds in the water-soluble fraction of *Salvia Miltiorrhiza*. *Salvianolic acid B* has been shown to protect endothelial cells and pericytes from inflammation, oxidative stress, and apoptosis, which delayed atherogenesis [33]. In addition, Sal B improved vascular function by inhibiting the inflammatory response and promoting endothelium-dependent vasodilation [35]. Some studies have indicated that Sal B may protect against AS by acting on MMPs. Jiang et al. [36] showed that Sal B inhibited MMP9, which prevented cardiac remodeling. In another study, Sal B significantly attenuated the expression of MMP9 and MMP12, and inhibited LPS-induced cell migration through inactivation of MMP2 and MMP9 protein synthesis [37].

Matrix metalloproteinases (MMPs) belong to the metzincin protease superfamily of zinc-dependent endopeptidases [38], and are associated with thinning of the fibrous cap and plaque instability in atherosclerosis [39, 40]. Matrix metalloproteinase 9(MMP9), also known as gelatinase B, has been widely studied in cardiovascular disease due to its association with plaque instability [41–43]. A study showed that lesion-targeted MMP9 inhibition ma promote plaque stabilization [44]. The expression of MMP9 in serum was positively associated with total carotid artery plaque score, larger intima-media thickness (IMT) value, and plaque instability [45]. Macrophage-derived MMP9 contributed to infiltration of monocytes and macrophages into lesions, but only overexpression of the autoactivating form of MMP9 in macrophages induced significant plaque disruption [39, 46]. In addition, a number of single nucleotide polymorphisms (SNPs) of the MMP9 gene are present in the promoter, coding, and untranslated regions, which may be of further interest. Gene analysis in a sample of 584 male patients showed a relationship between the 1562C > T polymorphism in the MMP9 gene and coronary heart disease severity [47]. Interestingly, one study showed no association between this MMP9 gene polymorphism and coronary heart disease [48], which suggests that further characterization of MMP9 polymorphism is needed. Previous studies in animals and humans have shown that MMP12 exerted atherosclerotic effects [49, 50]. A genome-wide association study showed that MMP12 was involved in large-artery atherosclerotic stroke through enhanced elastin degradation and macrophage invasion in plaques [51]. In addition, MMP12 and HDAC9 gene expression levels were associated with risk of ischemic stroke, and inhibition of the expression of these genes inhibited plaque development and promoted plaque stability [52]. Phosphinic peptide (RXP470.1), a selective murine MMP12 inhibitor, significantly reduced atherosclerotic plaque cross-sectional area in male and female Apoe knockout mice [53]. Analyses of visceral and subcutaneous white adipose tissue from mice and humans showed that MMP12 was upregulated in obesity at the mRNA and protein levels, which resulted in increased activity; this indicated that MMP12 may be a target for treatment and prevention of cardiometabolic diseases [54]. Andrie et al. [55] suggested that the effect of common MMP genotypes on plaque formation may be site- and sex-dependent, which was reflected by the association of with the MMP9 279Q allele with plaques in men and the association of the MMP12 82G allele with plaques in women. These associations between MMP9 or MMP12 and cardiovascular disease indicate that more basic research and clinical studies are needed to confirm the diagnostic significance and therapeutic potential of MMP9 and MMP12.

Sal B has been proved to have inhibitory effects on immunity. A study on experimental autoimmune encephalomyelitis showed that Sal B reduced inflammatory cell infiltration, and inhibited Th1 cell responses [56]. Sal B inhibited ox-LDL-induced maturation of human monocyte-derived dendritic cells through PPARγ activation,which might reduce inflammatory and immune responses [57]. Blockade of toll-like receptor 4 (TLR4), a receptor related to innate immunity and inflammatory signaling, by Sal B resulted in downregulation of NF-κB transcriptional activity and inhibiting immune responses [58]. Although researchers pointed out that Sal B is a small compounds with multiple mechanisms for cardiovascular protection [59], there are few studies on the exact immune mechanisms of Sal B on AS. Antigen presentation and recognition in mice is much simpler than in humans because of control of experimental conditions. Characterization of the effects on regulating immunity of Sal B in atherosclerosis with bioinformatic analysis,further basic experiments, is of great potential value.

To study the role of immune cell infiltration in the progression of AS, CIBERSORT and ssGSEA was used to characterize immune infiltration in AS progression. Patients with advanced atherosclerotic plaques had higher proportions of B cells memory, Plasma cells, T cells CD4 memory activated, T cells gamma delta, and Macrophages M0, and lower proportions of B cells naive, T cells CD8, T cells CD4 memory resting, T cells regulatory (Tregs), NK cells activated, and Monocytes than those in patients with early atherosclerotic plaques. These results have been verified by ssGSEA, indicating that these immune cells are involving in progression of AS. Atherosclerosis is a multifactorial disease characterized by immune-inflammatory remodeling of the arterial walls. Macrophages contribute to maintenance of the local inflammatory response, and can propagate plaque development and promote thrombosis [60]. Macrophage polarization is critical to the inflammatory response, as M1 macrophages initiate and sustain inflammation, and activated M2 macrophages resolve inflammation [61]. A study showed local and systemic B cell responses in response to a cholesterol-rich diet, which indicated activation of the innate immune response in atherosclerosis [62]. Activated CD4^+^ T cells were detected in atherosclerotic plaques of 57 patients with critical stenosis of the carotid subjected to endarterectomy [63], which indicate typical immune cells participation in disease progression and destabilization. Treg subset seems to represent a specific cellular pattern displayed by patients with symptomatic carotid artery stenosis and associated with brain injury [64]. Although many studies have investigated the inflammatory-immune relationship in atherosclerosis in murine models, significant challenges remain for translation of animal research to clinical use.

We showed that MMP9 and MMP12 were positively correlated with M0 macrophage and gamma delta T cell infiltration, and negatively correlated with Tregs, CD8^+^ T cell, and activated NK cell infiltration. A previous study showed that MMPs could induce immune responses through degradation of extracellular matrix proteins [65]. Metastasis-associated lung adenocarcinoma transcript 1 (MALAT1) is involved in cardiac innate immunity in a myocarditis model, and the expression of MMP9 was decreased in MALAT1-deficient bone marrow-derived macrophages [66]. In human promonocytic U937 cells, upregulation of MMP9 via TLR4, a key receptor in innate immunity, may sustain the inflammatory response and matrix degradation, resulting in atherosclerotic plaque instability [67]. A clinical study suggested that Th17 cells were related to the late stages of carotid artery stenosis, but not to plaque instability. Moreover, Tregs seem to represent a cellular pattern and were associated with brain injury [64]. More studies are needed to characterize the complex interactions between genes and immune cells.

To identify changes happening at the transcriptional level, we used a network-based approach to explore the relationship between MMP9 and MMP12 with miRNAs and TFs. Results show that miR-34a-5p might be the most important microRNA target, meanwhile, FOXC1 and JUN might be most highly correlated TFs with both candidated genes. Down-regulation of miR-34a-5p have been proved to increase protective effect against ischemic myocardial infarction by stimulating the expression of MMP9 [68]. On the foundation of bioinformatic analysis, more studies are needed to explore the molecular mechanisms and functions of immune cell infiltration in atherosclerosis.

This study identified MMP9 and MMP12 as candidate genes for the anti-AS effects of Sal B, and explored the associations among AS plaque stability, Sal B target genes, and immune cell infiltration. However, our study was subject to some limitations. First, we used data from the GEO public database, and the number of samples was limited. A greater number of clinical samples will be used in future studies. Second, we need to further study and explore the underlying mechanisms of candidate genes associated with AS plaque stability and immune cell infiltration, and further characterize of the anti-AS effects of Sal B.

## Conclusions

Using bioinformatics analysis, we identified MMP9 and MMP12 as candidate genes through which Sal B may stabilize AS plaques. We verified the reliability of these two genes using ROC curves and molecular docking analysis. The results suggested that MMP9 and MMP12 may be potential targets for Sal B intervention in ASCVD. In addition, we showed that B cells naive, B cells memory, Plasma cells, T cells CD8, T cells CD4 memory resting, T cells CD4 memory activated, T cells regulatory (Tregs), T cells gamma delta, NK cells activated, Monocytes, and Macrophages M0 may be involved in AS progression. Furthermore, MMP9 and MMP12 were associated with these immune cells to different degrees. Meanwhile, miR-34a-5p and FOXC1, JUN maybe the most important miRNA and TFs for Sal B anti-AS. Further evaluation of the roles of these immune cells in AS may result in novel targeted immunotherapies for early AS intervention.

## Supplementary Information

**Additional file 1: Supplementary Table 1.** Sal B-related targets.

**Additional file 2: Supplementary Table 2.** Differentially expressed genes in GSE28829 and GSE43292.

**Additional file 3: Supplementary Table 3.** Topology analysis results.

**Additional file 4: Supplementary Table 4.** GO analysis results.

**Additional file 5: Supplementary Table 5.** GSEA results.

**Additional file 6: Supplementary Table 6.** Turquoise module genes of WGCNA in GSE28829 and GSE43292.

## Data Availability

The datasets of target of Sal B analysed during the current study are available in the SEA (http://sea.bkslab.org/) and SIB (https://www.sib.swiss/) repository. The datasets (GSE28829 and GSE43292) for this study come from The Gene Expression Omnibus (GEO) database (https://www.ncbi.nlm.nih.gov/geo/). All the data in this paper support the results of this study, Other datasets used and/or analyzed during the current study are available from the corresponding author on reasonable request.

## Abbreviations

AS: Atherosclerosis
ASCVD: Atherosclerotic cardiovascular disease
Sal B: Salvianolic acid B
DEGs: Differentially expressed genes
WGCNA: Weighted gene co-expression network analysis
ROC: Receiver operating characteristic
MI: Myocardial infarction
Tregs: Regulatory T cells
VSMC: Vascular smooth muscle cell
PPI: Protein–protein interaction
GO: Gene Ontology
GSEA: Gene Set Enrichment Analysis
AUC: Area under the curve
SEA: Similarity ensemble approach
SIB: Swiss Institute of Bioinformatics
MMPs: Matrix metalloproteinases
SNPs: Single nucleotide polymorphisms
