# Supplementary material for: Identification of core genes associated with the anti-atherosclerotic effects of Salvianolic acid B and immune cell infiltration characteristics using bioinformatics analysis
Source: BMC Complement Med Ther. 2022 Jul 16;22:190. doi: 10.1186/s12906-022-03670-6 (PMC9288713; doi:10.1186/s12906-022-03670-6)
Supplement: Supplementary file 1 — Additional file 1: Supplementary Table 1. Sal B-related targets. [file 12906_2022_3670_MOESM1_ESM.pdf]

Gene ID						
ACPM	AK1BA	AK1C4	CIA30	JUN	MMP12	NDUA1
NDUA2	NDUA4	NDUA3	NDUA5	NDUA6	NDUA8	NDUA7
NDUA9	NDUAA	NDUAB	NDUAC	NDUAD	NDUB1	NDUB2
NDUB3	NDUB4	NDUB5	NDUB6	NDUB7	NDUB8	NDUB9
NDUBA	NDUBB	NDUC1	NDUC2	NDUF2	NDUF3	NDUF4
NDUS2	NDUS1	NDUS3	NDUS4	NDUS5	NDUS6	NDUS7
NDUS8	NDUV1	NDUV2	NDUV3	NU1M	NU2M	NU3M
NU4LM	NU4M	NU5M	NU6M	NUA4L	TTHY	XDH
AKR1B1	CA7	CA12	CA4	MMP9	MMP1	APP
MMP3						

Supplementary Table 1 : Sal B-related targets.
