# Supplementary material for: Identification of core genes associated with the anti-atherosclerotic effects of Salvianolic acid B and immune cell infiltration characteristics using bioinformatics analysis
Source: BMC Complement Med Ther. 2022 Jul 16;22:190. doi: 10.1186/s12906-022-03670-6 (PMC9288713; doi:10.1186/s12906-022-03670-6)
Supplement: Supplementary file 2 — Additional file 2: Supplementary Table 2. Differentially expressed genes in GSE28829 and GSE43292. [file 12906_2022_3670_MOESM2_ESM.pdf]

Gene ID	logFC	AveExpr	t	P.Value	adj.P.Val	B
CCR1	1.298509	7.500535	8.271192	7.92E-13	1.69E-09	18.81878
ATP1A2	-1.05929	7.164645	-8.10046	1.82E-12	2.29E-09	18.01666
ANGPTL1	-1.13263	6.508069	-8.06391	2.17E-12	2.55E-09	17.84532
PIK3AP1	1.019671	7.984392	7.966977	3.48E-12	2.71E-09	17.39142
ACADL	-1.25254	6.822923	-7.94979	3.78E-12	2.71E-09	17.31105
CNTN4	-1.5051	7.181953	-7.92446	4.28E-12	2.71E-09	17.19265
SLAMF8	1.113726	7.566698	7.902788	4.75E-12	2.80E-09	17.09139
ITGB2	1.137052	8.996515	7.887588	5.12E-12	2.80E-09	17.0204
TYROBP	1.074293	9.793521	7.882305	5.25E-12	2.80E-09	16.99574
C1QA	1.030128	8.917836	7.875217	5.43E-12	2.82E-09	16.96265
CNN1	-1.12136	9.75182	-7.8295	6.78E-12	3.28E-09	16.74935
NEXN	-1.03163	9.809091	-7.80199	7.74E-12	3.33E-09	16.62112
CD52	1.456658	7.749204	7.730081	1.10E-11	4.02E-09	16.28639
NPL	1.080814	7.656546	7.563868	2.44E-11	6.82E-09	15.51513
CD14	1.099317	10.186	7.525744	2.93E-11	7.48E-09	15.33875
C1QB	1.068492	9.525999	7.519355	3.02E-11	7.60E-09	15.30921
VAMP8	1.166045	8.977992	7.476392	3.71E-11	8.47E-09	15.11074
PLEK	1.07629	8.68211	7.468418	3.86E-11	8.60E-09	15.07392
LAPTM5	1.026773	11.29236	7.36641	6.28E-11	1.15E-08	14.6039
ITGAM	1.148229	8.344817	7.328768	7.52E-11	1.30E-08	14.43086
PLD5	-1.0758	5.914579	-7.29735	8.73E-11	1.41E-08	14.28661
CD180	1.000317	6.969747	7.291308	8.99E-11	1.41E-08	14.2589
FIBIN	-1.06437	9.224501	-7.25893	1.05E-10	1.58E-08	14.11042
C1QC	1.059954	9.510811	7.243822	1.13E-10	1.64E-08	14.04123
TMEM56	-1.01165	6.630992	-7.19065	1.45E-10	1.88E-08	13.79794
LY86	1.090146	8.076082	7.188379	1.47E-10	1.88E-08	13.78757
CASQ2	-1.50121	8.603447	-7.1832	1.50E-10	1.88E-08	13.76393
GIMAP2	1.004885	7.189681	7.085867	2.38E-10	2.50E-08	13.32001
PRUNE2	-1.0104	8.905385	-6.99877	3.59E-10	3.22E-08	12.92428
FCER1G	1.107927	9.318292	6.998411	3.60E-10	3.22E-08	12.92266
CTSS	1.044957	9.557625	6.995206	3.65E-10	3.23E-08	12.90813
PLCB4	-1.03288	6.290369	-6.98703	3.80E-10	3.25E-08	12.87107
ACP5	1.314745	7.856393	6.924439	5.10E-10	3.91E-08	12.58774
FABP5	1.535886	8.772393	6.887284	6.07E-10	4.33E-08	12.41992
MYOCD	-1.41362	8.376331	-6.83444	7.77E-10	5.13E-08	12.18175

CPVL	1.044576	8.454671	6.813949	8.55E-10	5.51E-08	12.08955
IGJ	2.145547	8.80372	6.763586	1.08E-09	6.49E-08	11.86333
NCF2	1.065154	8.555592	6.732219	1.25E-09	7.25E-08	11.72272
PLTP	1.02511	10.17499	6.712294	1.37E-09	7.73E-08	11.63351
MS4A4A	1.139613	8.032544	6.634743	1.97E-09	9.98E-08	11.28718
CNTN1	-1.44237	6.321904	-6.63313	1.98E-09	9.99E-08	11.28
CD163	1.09351	9.845784	6.567811	2.68E-09	1.26E-07	10.9894
FHL5	-1.10749	7.516773	-6.51991	3.35E-09	1.47E-07	10.77697
SNX10	1.06737	7.173223	6.499369	3.68E-09	1.60E-07	10.68604
CYTIP	1.194512	7.846067	6.489947	3.84E-09	1.64E-07	10.64437
FBP1	1.045536	7.701316	6.461808	4.38E-09	1.80E-07	10.52005
FCGR2B	1.088312	8.78335	6.404286	5.70E-09	2.20E-07	10.26655
HAND2-AS1	-1.28675	6.186631	-6.3958	5.92E-09	2.25E-07	10.22923
HMOX1	1.290539	8.138336	6.360655	6.95E-09	2.57E-07	10.07484
PLA2G7	1.260021	7.810262	6.340493	7.62E-09	2.76E-07	9.986418
CNTN3	-1.23473	6.98742	-6.33519	7.81E-09	2.81E-07	9.963184
IBSP	1.287277	6.848619	6.325738	8.16E-09	2.90E-07	9.921783
MMP9	1.881409	8.674188	6.318281	8.44E-09	2.94E-07	9.889137
PDE8B	-1.07579	6.492667	-6.27703	1.02E-08	3.40E-07	9.70885
MRAP2	-1.02602	9.468244	-6.23575	1.23E-08	3.85E-07	9.528875
LRRN1	-1.03643	4.947382	-6.19562	1.47E-08	4.41E-07	9.354375
IGKC	1.352779	7.768893	6.131913	1.96E-08	5.36E-07	9.078343
TPH1	-1.56668	7.823477	-6.11554	2.11E-08	5.69E-07	9.00759
CCL8	1.016269	7.236023	6.113915	2.13E-08	5.70E-07	9.000573
MMRN1	1.152795	5.82	6.00453	3.48E-08	8.49E-07	8.530016
CLEC5A	1.089551	7.135091	5.984575	3.80E-08	9.08E-07	8.444567
C15orf48	1.023106	5.740978	5.932954	4.78E-08	1.08E-06	8.22411
ADAMDEC1	1.367907	6.127742	5.927172	4.91E-08	1.10E-06	8.19947
CXCR4	1.020409	9.282282	5.714871	1.25E-07	2.32E-06	7.302388
CCL19	1.302535	7.611763	5.674664	1.49E-07	2.67E-06	7.134227
SPP1	1.139331	11.07226	5.59073	2.15E-07	3.56E-06	6.785034
DPP4	1.081479	6.635512	5.558165	2.48E-07	3.99E-06	6.650244
CYP1B1	1.014687	8.066463	5.484909	3.40E-07	5.17E-06	6.348471
TREM1	1.0973	7.848149	5.460854	3.76E-07	5.62E-06	6.24982
AQP9	1.356904	8.327239	5.422865	4.43E-07	6.43E-06	6.094479
ATRNL1	-1.08182	7.124068	-5.30131	7.43E-07	9.68E-06	5.601239

CHI3L1	1.320499	8.287713	5.249652	9.23E-07	1.15E-05	5.393411
CD36	1.371417	8.60651	5.216164	1.06E-06	1.28E-05	5.259276
FABP4	1.54713	6.352861	5.051184	2.11E-06	2.25E-05	4.605395
CCL18	1.243731	9.644676	5.028988	2.31E-06	2.43E-05	4.518327
MMP12	1.847879	8.345669	4.903937	3.86E-06	3.72E-05	4.031916
MMP7	1.412399	6.889807	4.85007	4.80E-06	4.43E-05	3.824603
RGS1	1.091792	7.844521	4.758065	6.95E-06	6.02E-05	3.473675
SELE	1.078793	5.967455	4.745692	7.30E-06	6.28E-05	3.42679
ITLN1	-1.24932	6.908674	-4.20347	5.93E-05	0.000365	1.449126
CARTPT	-1.01674	6.370679	-3.39764	0.000993	0.004085	-1.17518

Supplementary Table 2: Differentially expressed genes in GSE28829 and GSE43292.
