# Supplementary material for: Identification of core genes associated with the anti-atherosclerotic effects of Salvianolic acid B and immune cell infiltration characteristics using bioinformatics analysis
Source: BMC Complement Med Ther. 2022 Jul 16;22:190. doi: 10.1186/s12906-022-03670-6 (PMC9288713; doi:10.1186/s12906-022-03670-6)
Supplement: Supplementary file 3 — Additional file 3: Supplementary Table 3. Topology analysis results. [file 12906_2022_3670_MOESM3_ESM.pdf]

name	Betweenness	Closeness	Degree	Eigenvector	Information	LAC	Network
ACADL	0	0.134293	1	3.40E-04	1.480696	0	0
FABP4	296.8048	0.153005	6	0.005655	3.023461	0.666667	2.25
ACP5	16.26329	0.165192	6	0.057382	3.023461	3.333333	4.6
IBSP	0	0.154696	3	0.01671	2.303456	2	3
TYROBP	95.80249	0.175549	24	0.256691	4.31968	12.58333	20.139
CTSS	115.4675	0.17284	19	0.198149	4.146835	9.157895	12.34904
MMP9	336.8304	0.176101	23	0.169489	4.289879	6.869565	16.57418
SPP1	256.6997	0.168675	11	0.051787	3.658829	3.272727	6
ITGAM	273.8305	0.180064	27	0.26634	4.398248	11.92593	22.03252
ADAMDEC1	0	0.153425	4	0.041992	2.591448	3	4
CCL18	76.60575	0.168675	16	0.136812	4.00542	6.75	10.64487
C1QA	68.02659	0.17284	21	0.232238	4.223654	11.90476	16.65535
C1QC	59.1885	0.172308	20	0.225962	4.186723	11.9	15.77552
SLAMF8	9.28599	0.164223	9	0.107479	3.455504	6.222222	7.625
AQP9	2.589194	0.16092	7	0.079888	3.189628	4.571429	5.333333
CCR1	57.87882	0.173913	21	0.238841	4.223654	12.38095	16.46569
CLEC5A	2.085901	0.16185	8	0.100392	3.33206	6	6.928571
PLEK	323.9696	0.171254	20	0.207276	4.186723	10.2	13.77676
CD14	140.3195	0.173913	19	0.207731	4.146835	10.31579	13.91315
TREM1	21.6171	0.169697	14	0.168047	3.887577	9.714286	11.46154

NCF2	43.54911	0.169697	14	0.158344	3.887577	8.285714	9.653846
ATP1A2	0	0.017857	1	0	1.480696	0	0
CNTN4	0	0.017857	1	0	1.480696	0	0
FCER1G	124.9731	0.174455	19	0.223531	4.146835	12.31579	15.00851
LAPTM5	13.63738	0.168675	16	0.191904	4.00542	11.25	13.56731
CCL19	8.314465	0.165192	11	0.121585	3.658829	7.090909	8.241667
C1QB	37.64201	0.172308	20	0.22987	4.186723	12.2	15.61105
MS4A4A	2.33192	0.163743	9	0.11737	3.455504	7.111111	8
FCGR2B	22.61165	0.171779	18	0.220935	4.103626	12.33333	14.06318
CD163	256.1025	0.175549	21	0.204592	4.223654	9.238095	13.78963
RGS1	6.814646	0.164223	9	0.111347	3.455504	5.777778	6.5
CCL8	3.16061	0.164223	9	0.099795	3.455504	6.222222	7
LY86	145.3515	0.170732	20	0.21565	4.186723	10.9	14.92427
ITGB2	112.8306	0.176101	25	0.262487	4.347558	12.48	20.76878
CASQ2	0	0.017857	1	0	1.480696	0	0
NEXN	0	0.017857	1	0	1.480696	0	0
CHI3L1	2.88473	0.159091	4	0.033704	2.591448	2	2.666667
MMP7	4.879519	0.163743	5	0.03986	2.827088	2.4	3
CXCR4	102.2429	0.169184	14	0.10935	3.887577	4.714286	7.562179
MMP12	4.412853	0.164223	5	0.049235	2.827088	2.8	3.5
SELE	7.595951	0.164706	7	0.062002	3.189628	4.571429	5.333333

CD180	0	0.156863	3	0.037128	2.303456	2	3
CD36	234.2943	0.162319	5	0.038499	2.827088	1.6	2.25
HMOX1	106.76	0.16568	7	0.054815	3.189628	2.857143	3.333333
PLA2G7	102	0.156863	3	0.022474	2.303456	0.666667	1
FABP5	23.44151	0.145455	3	0.002674	2.303456	1.333333	2.5
CD52	2.213889	0.162319	8	0.100671	3.33206	6	7.028571
CYTIP	4.689335	0.16092	6	0.063922	3.023461	3.333333	4
CNN1	102	0.148936	2	0.012419	1.943485	0	0
MYOCD	0	0.131148	1	7.40E-04	1.480696	0	0
CPVL	0	0.134615	2	5.01E-04	1.943485	1	2
DPP4	0	0.153425	2	0.016707	1.943485	1	2
CYP1B1	0	0.143959	1	0.003287	1.480696	0	0
ITLN1	0	0.134293	1	3.41E-04	1.480696	0	0
LRRN1	0	0.147757	1	0.012876	1.480696	0	0
PIK3AP1	0	0.148148	1	0.012375	1.480696	0	0
PLTP	0	0.137255	1	0.001348	1.480696	0	0

Supplementary Table 3 : Topology analysis.
