# Supplementary material for: Identification of core genes associated with the anti-atherosclerotic effects of Salvianolic acid B and immune cell infiltration characteristics using bioinformatics analysis
Source: BMC Complement Med Ther. 2022 Jul 16;22:190. doi: 10.1186/s12906-022-03670-6 (PMC9288713; doi:10.1186/s12906-022-03670-6)
Supplement: Supplementary file 4 — Additional file 4: Supplementary Table 4. GO analysis results. [file 12906_2022_3670_MOESM4_ESM.pdf]

ID	Description	qvalue	geneID	Count
0050900	leukocyte migration	2.98E-07	CCR1/SLAMF8/ITGB2/FCER1G/HMOX1/PLA2G7/MMP9/CCL8/CXCR4/CCL19/DPP4/TREM1/ CCL18/SELE	14
0030595	leukocyte chemotaxis	1.40E-06	CCR1/SLAMF8/ITGB2/FCER1G/PLA2G7/CCL8/CXCR4/CCL19/DPP4/TREM1/CCL18	11
0097529	myeloid leukocyte migration	8.77E-06	CCR1/SLAMF8/ITGB2/FCER1G/PLA2G7/CCL8/CCL19/DPP4/TREM1/CCL18	10
1990266	neutrophil migration	1.14E-05	SLAMF8/ITGB2/FCER1G/CCL8/CCL19/DPP4/TREM1/CCL18	8
0060326	cell chemotaxis	1.24E-05	CCR1/SLAMF8/ITGB2/FCER1G/PLA2G7/CCL8/CXCR4/CCL19/DPP4/TREM1/CCL18	11
0098883	synapse pruning	1.93E-05	C1QA/C1QB/ITGAM/C1QC	4
0002886	regulation of myeloid leukocyte mediated immunity	2.02E-05	ITGB2/TYROBP/VAMP8/ITGAM/FCGR2B/HMOX1	6
0097530	granulocyte migration	2.32E-05	SLAMF8/ITGB2/FCER1G/CCL8/CCL19/DPP4/TREM1/CCL18	8
0002444	myeloid leukocyte mediated immunity	2.32E-05	ITGB2/TYROBP/VAMP8/ITGAM/FCGR2B/HMOX1/TREM1	7
0002443	leukocyte mediated immunity	2.49E-05	ITGB2/TYROBP/C1QA/C1QB/VAMP8/ITGAM/C1QC/FCER1G/FCGR2B/HMOX1/IGKC/TREM1	12
0030593	neutrophil chemotaxis	2.49E-05	ITGB2/FCER1G/CCL8/CCL19/DPP4/TREM1/CCL18	7
0042119	neutrophil activation	3.58E-05	ITGB2/TYROBP/ITGAM/FCER1G/FCGR2B	5
	regulation of superoxide	3.58E-05	ITGB2/TYROBP/ITGAM/ACP5/CD36	5

0090322	metabolic process			
0002548	monocyte chemotaxis	3.89E-05	CCR1/SLAMF8/PLA2G7/CCL8/CCL19/CCL18	6
0006801	superoxide metabolic process	5.07E-05	ITGB2/TYROBP/ITGAM/ACP5/NCF2/CD36	6
0071621	granulocyte chemotaxis	6.19E-05	ITGB2/FCER1G/CCL8/CCL19/DPP4/TREM1/CCL18	7
0002283	neutrophil activation involved in immune response	6.19E-05	ITGB2/TYROBP/ITGAM/FCER1G	4
0036230	granulocyte activation	6.47E-05	ITGB2/TYROBP/ITGAM/FCER1G/FCGR2B	5
0042554	superoxide anion generation	6.89E-05	ITGB2/TYROBP/ITGAM/ACP5/NCF2	5
0002697	regulation of immune effector process	7.32E-05	SLAMF8/ITGB2/TYROBP/VAMP8/LAPTM5/ITGAM/FCGR2B/HMOX1/CCL19/CD36	10
0043300	regulation of leukocyte degranulation	7.82E-05	ITGB2/VAMP8/ITGAM/FCGR2B/HMOX1	5
0150146	cell junction disassembly	9.27E-05	C1QA/C1QB/ITGAM/C1QC	4
0022411	cellular component disassembly	9.47E-05	C1QA/C1QB/VAMP8/PLEK/ITGAM/C1QC/CTSS/MMP9/DPP4/MMP12/MMP7	11
0002366	leukocyte activation involved in immune response	9.47E-05	ITGB2/TYROBP/VAMP8/ITGAM/CD180/FCER1G/FCGR2B/HMOX1/CCL19	9
0002263	cell activation involved in immune response	9.98E-05	ITGB2/TYROBP/VAMP8/ITGAM/CD180/FCER1G/FCGR2B/HMOX1/CCL19	9
	myeloid cell activation	9.98E-05	ITGB2/TYROBP/VAMP8/ITGAM/FCER1G/HMOX1	6

0002275	involved in immune response			
0002685	regulation of leukocyte migration	0.000109	CCR1/SLAMF8/HMOX1/PLA2G7/CCL8/CCL19/DPP4/SELE	8
0032928	regulation of superoxide anion generation	0.000128	ITGB2/TYROBP/ITGAM/ACP5	4
0002274	myeloid leukocyte activation	0.000158	ITGB2/TYROBP/C1QA/VAMP8/ITGAM/FCER1G/FCGR2B/HMOX1	8
0002407	dendritic cell chemotaxis	0.000167	CCR1/SLAMF8/CXCR4/CCL19	4
0006909	phagocytosis	0.000186	ITGB2/TYROBP/CD14/ITGAM/FCER1G/NCF2/FCGR2B/IGKC/CD36	9
0002699	positive regulation of immune effector process	0.000211	ITGB2/TYROBP/VAMP8/LAPTM5/ITGAM/HMOX1/CCL19/CD36	8
0022617	extracellular matrix disassembly	0.000242	CTSS/MMP9/DPP4/MMP12/MMP7	5
0006898	receptor-mediated endocytosis	0.000262	ITGB2/CD14/ITGAM/FCER1G/FCGR2B/CCL19/CD36/SELE	8
0032103	positive regulation of response to external stimulus	0.000323	CCR1/TYROBP/VAMP8/CD180/LY86/PLA2G7/CXCR4/CCL19/FABP4/MMP12	10
0034612	response to tumor necrosis factor	0.000323	CD14/LAPTM5/CCL8/CCL19/CHI3L1/FABP4/CCL18/SELE	8
0036336	dendritic cell migration	0.000364	CCR1/SLAMF8/CXCR4/CCL19	4
0043299	leukocyte degranulation	0.000433	ITGB2/VAMP8/ITGAM/FCGR2B/HMOX1	5
	exocytosis	0.000433	CCR1/ITGB2/VAMP8/PLEK/ITGAM/FCGR2B/HMOX1/TPH1/CCL8	9

0006887				
1902563	regulation of neutrophil activation	0.000496	ITGB2/ITGAM/FCGR2B	3
0071674	mononuclear cell migration	0.000496	CCR1/SLAMF8/PLA2G7/CCL8/CXCR4/CCL19/CCL18	7
0097242	amyloid-beta clearance	0.000569	ITGB2/ITGAM/MYOCD/CD36	4
0001819	positive regulation of cytokine production	0.000578	TYROBP/CD14/LAPTM5/FCER1G/HMOX1/CCL19/CYP1B1/CHI3L1/CD36/MMP12	10
0010038	response to metal ion	0.000608	C1QA/CD14/FIBIN/CASQ2/FBP1/HMOX1/MMP9/AQP9/FABP4	9
0002573	myeloid leukocyte differentiation	0.000664	CCR1/TYROBP/C1QC/SNX10/MMP9/CCL19/CARTPT	7
0050727	regulation of inflammatory response	0.000762	PIK3AP1/SLAMF8/VAMP8/ACP5/FCGR2B/PLA2G7/MMP9/FABP4/SELE	9
0030574	collagen catabolic process	0.000762	CTSS/MMP9/MMP12/MMP7	4
0070098	chemokine-mediated signaling pathway	0.000858	CCR1/CCL8/CXCR4/CCL19/CCL18	5
0001774	microglial cell activation	0.001137	ITGB2/TYROBP/C1QA/ITGAM	4
0045055	regulated exocytosis	0.001137	ITGB2/VAMP8/PLEK/ITGAM/FCGR2B/HMOX1/TPH1	7
2000377	regulation of reactive oxygen species metabolic process	0.00117	ITGB2/TYROBP/ITGAM/ACP5/CYP1B1/CD36	6
0120162	positive regulation of cold-induced	0.001198	ACADL/FABP5/CXCR4/CD36/FABP4	5

	thermogenesis			
1901216	positive regulation of neuron death	0.001198	ITGB2/TYROBP/C1QA/ITGAM/FCGR2B	5
1990868	response to chemokine	0.001198	CCR1/CCL8/CXCR4/CCL19/CCL18	5
1990869	cellular response to chemokine	0.001198	CCR1/CCL8/CXCR4/CCL19/CCL18	5
0072593	reactive oxygen species metabolic process	0.001296	ITGB2/TYROBP/ITGAM/ACP5/NCF2/CYP1B1/CD36	7
0002888	positive regulation of myeloid leukocyte mediated immunity	0.001504	ITGB2/TYROBP/ITGAM	3
0002683	negative regulation of immune system process	0.001504	SLAMF8/TYROBP/LAPTM5/C1QC/FCGR2B/HMOX1/DPP4/MMP12/CARTPT	9
0042116	macrophage activation	0.001688	ITGB2/TYROBP/C1QA/ITGAM/FCGR2B	5
0032930	positive regulation of superoxide anion generation	0.001688	ITGB2/TYROBP/ITGAM	3
1904645	response to amyloid-beta	0.001846	FCGR2B/MMP9/CD36/MMP12	4
0071404	cellular response to low-density lipoprotein particle stimulus	0.002161	ITGB2/FCER1G/CD36	3
0055074	calcium ion homeostasis	0.002161	CCR1/ATP1A2/CD52/CASQ2/SNX10/CCL8/CXCR4/CCL19/CD36	9
0031663	lipopolysaccharide-mediated signaling	0.002308	CD14/CD180/LY86/CD36	4

	pathway			
0002764	immune response-regulating signaling pathway	0.002387	PIK3AP1/TYROBP/CD14/LAPTM5/FCER1G/CTSS/FCGR2B/IGKC/CD36	9
0051051	negative regulation of transport	0.002428	ATP1A2/VAMP8/CASQ2/FABP5/FCGR2B/HMOX1/MMP9/CD36/CARTPT	9
0032615	interleukin-12 production	0.00247	LAPTM5/ACP5/CCL19/CD36	4
0032655	regulation of interleukin-12 production	0.00247	LAPTM5/ACP5/CCL19/CD36	4
0043302	positive regulation of leukocyte degranulation	0.002577	ITGB2/VAMP8/ITGAM	3
0043410	positive regulation of MAPK cascade	0.002619	CCR1/LAPTM5/FCGR2B/CCL8/CCL19/CHI3L1/CD36/CCL18/CARTPT	9
0002455	humoral immune response mediated by circulating immunoglobulin	0.002619	C1QA/C1QB/C1QC/FCGR2B/IGKC	5
0002253	activation of immune response	0.002619	TYROBP/C1QA/C1QB/LAPTM5/C1QC/FCER1G/FCGR2B/IGKC	8
1902105	regulation of leukocyte differentiation	0.002619	CCR1/SLAMF8/TYROBP/C1QC/FCGR2B/CCL19/CARTPT	7
0002688	regulation of leukocyte chemotaxis	0.002649	CCR1/SLAMF8/PLA2G7/CCL19/DPP4	5
0031664	regulation of lipopolysaccharide-mediated signaling pathway	0.002658	CD180/LY86/CD36	3

0045453	bone resorption	0.002658	ACP5/SNX10/SPP1/CARTPT	4
0006911	phagocytosis, engulfment	0.003075	ITGB2/ITGAM/FCGR2B/IGKC/CD36	5
0019722	calcium-mediated signaling	0.003077	CCR1/ATP1A2/PLEK/CASQ2/CXCR4/SELE	6
0016064	immunoglobulin mediated immune response	0.00347	C1QA/C1QB/C1QC/FCER1G/FCGR2B/IGKC	6
0061082	myeloid leukocyte cytokine production	0.003556	LAPTM5/HMOX1/CD36	3
0019724	B cell mediated immunity	0.003658	C1QA/C1QB/C1QC/FCER1G/FCGR2B/IGKC	6
0030198	extracellular matrix organization	0.00372	CTSS/IBSP/MMP9/DPP4/CYP1B1/MMP12/MMP7	7
0043062	extracellular structure organization	0.003747	CTSS/IBSP/MMP9/DPP4/CYP1B1/MMP12/MMP7	7
0090025	regulation of monocyte chemotaxis	0.003747	CCR1/SLAMF8/PLA2G7	3
0045229	external encapsulating structure organization	0.003747	CTSS/IBSP/MMP9/DPP4/CYP1B1/MMP12/MMP7	7
0099024	plasma membrane invagination	0.003747	ITGB2/ITGAM/FCGR2B/IGKC/CD36	5
1903305	regulation of regulated secretory pathway	0.003747	ITGB2/VAMP8/ITGAM/FCGR2B/HMOX1	5
0019886	antigen processing and presentation of exogenous peptide	0.003974	FCER1G/CTSS/FCGR2B	3

	antigen via MHC class II			
0070374	positive regulation of ERK1 and ERK2 cascade	0.003974	CCR1/CCL8/CCL19/CHI3L1/CD36/CCL18	6
2000379	positive regulation of reactive oxygen species metabolic process	0.004114	ITGB2/TYROBP/ITGAM/CD36	4
0019932	second-messenger-mediated signaling	0.004172	CCR1/ATP1A2/PLEK/CASQ2/CXCR4/CD36/SELE	7
0050920	regulation of chemotaxis	0.004377	CCR1/SLAMF8/PLA2G7/CXCR4/CCL19/DPP4	6
0050867	positive regulation of cell activation	0.004377	ITGB2/TYROBP/VAMP8/PLEK/ITGAM/IGKC/CCL19/DPP4	8
0070555	response to interleukin-1	0.004377	CCL8/CCL19/CHI3L1/CCL18/SELE	5
0010324	membrane invagination	0.004381	ITGB2/ITGAM/FCGR2B/IGKC/CD36	5
0106106	cold-induced thermogenesis	0.004381	ACADL/FABP5/CXCR4/CD36/FABP4	5
0120161	regulation of cold-induced thermogenesis	0.004381	ACADL/FABP5/CXCR4/CD36/FABP4	5
0008037	cell recognition	0.004385	CNTN4/NEXN/IGKC/CXCR4/CCL19/CD36	6
0007204	positive regulation of cytosolic calcium ion concentration	0.004387	CCR1/ATP1A2/CD52/CASQ2/CXCR4/CCL19/CD36	7
0002703	regulation of leukocyte mediated immunity	0.0044	ITGB2/TYROBP/VAMP8/ITGAM/FCGR2B/HMOX1	6
	cellular response to tumor	0.004675	LAPTM5/CCL8/CCL19/CHI3L1/FABP4/CCL18	6

0071356	necrosis factor			
0002831	regulation of response to biotic stimulus	0.004818	SLAMF8/TYROBP/CD180/LY86/FCGR2B/CD36/MMP12	7
0002446	neutrophil mediated immunity	0.004818	ITGB2/ITGAM/TREM1	3
0002495	antigen processing and presentation of peptide antigen via MHC class II	0.004818	FCER1G/CTSS/FCGR2B	3
0050869	negative regulation of B cell activation	0.004818	TYROBP/LAPTM5/FCGR2B	3
0055094	response to lipoprotein particle	0.004818	ITGB2/FCER1G/CD36	3
0097006	regulation of plasma lipoprotein particle levels	0.005315	PLTP/HMOX1/PLA2G7/CD36	4
0002504	antigen processing and presentation of peptide or polysaccharide antigen via MHC class II	0.005559	FCER1G/CTSS/FCGR2B	3
0071402	cellular response to lipoprotein particle stimulus	0.005559	ITGB2/FCER1G/CD36	3
1990845	adaptive thermogenesis	0.005705	ACADL/FABP5/CXCR4/CD36/FABP4	5
0006874	cellular calcium ion homeostasis	0.005705	CCR1/ATP1A2/CD52/CASQ2/CCL8/CXCR4/CCL19/CD36	8
0045730	respiratory burst	0.00587	SLAMF8/CD52/NCF2	3
	bone remodeling	0.006189	ACP5/SNX10/SPP1/CARTPT	4

0046849				
1904705	regulation of vascular associated smooth muscle cell proliferation	0.006189	CNN1/MYOCD/HMOX1/MMP9	4
0002478	antigen processing and presentation of exogenous peptide antigen	0.006189	FCER1G/CTSS/FCGR2B	3
1990874	vascular associated smooth muscle cell proliferation	0.006355	CNN1/MYOCD/HMOX1/MMP9	4
0051480	regulation of cytosolic calcium ion concentration	0.006814	CCR1/ATP1A2/CD52/CASQ2/CXCR4/CCL19/CD36	7
0043112	receptor metabolic process	0.006913	ITGB2/LAPTM5/FCER1G/CD36/SELE	5
0030316	osteoclast differentiation	0.006986	CCR1/TYROBP/SNX10/CARTPT	4
0002460	adaptive immune response based on somatic recombination of immune receptors built from immunoglobulin superfamily domains	0.006986	C1QA/C1QB/C1QC/FCER1G/FCGR2B/IGKC/CCL19	7
0032735	positive regulation of interleukin-12 production	0.007263	LAPTM5/CCL19/CD36	3
1902622	regulation of neutrophil migration	0.007263	SLAMF8/CCL19/DPP4	3
	cytokine-mediated	0.007263	CCR1/LAPTM5/FCER1G/CCL8/CXCR4/CCL19/CCL18/MMP12	8

0019221	signaling pathway			
0002237	response to molecule of bacterial origin	0.007586	CD14/CD180/LY86/ACP5/FCGR2B/CD36/SELE	7
0002367	cytokine production involved in immune response	0.007804	LAPTM5/HMOX1/TREM1/CD36	4
1903706	regulation of hemopoiesis	0.007951	CCR1/SLAMF8/TYROBP/C1QC/FCGR2B/CCL19/CARTPT	7
0001659	temperature homeostasis	0.007951	ACADL/FABP5/CXCR4/CD36/FABP4	5
1904706	negative regulation of vascular associated smooth muscle cell proliferation	0.007991	CNN1/MYOCD/HMOX1	3
0045807	positive regulation of endocytosis	0.007991	CD14/CCL19/CD36/SELE	4
0002887	negative regulation of myeloid leukocyte mediated immunity	0.007991	FCGR2B/HMOX1	2
0070391	response to lipoteichoic acid	0.007991	CD14/CD36	2
0071223	cellular response to lipoteichoic acid	0.007991	CD14/CD36	2
0072503	cellular divalent inorganic cation homeostasis	0.00813	CCR1/ATP1A2/CD52/CASQ2/CCL8/CXCR4/CCL19/CD36	8
0032760	positive regulation of tumor necrosis factor	0.008777	TYROBP/CD14/CCL19/CD36	4

	production			
0032640	tumor necrosis factor production	0.008869	TYROBP/CD14/ACP5/CCL19/CD36	5
0032680	regulation of tumor necrosis factor production	0.008869	TYROBP/CD14/ACP5/CCL19/CD36	5
0032963	collagen metabolic process	0.008901	CTSS/MMP9/MMP12/MMP7	4
0030099	myeloid cell differentiation	0.00906	CCR1/TYROBP/C1QC/SNX10/MMP9/CCL19/CARTPT	7
0002604	regulation of dendritic cell antigen processing and presentation	0.00912	FCGR2B/CCL19	2
0032493	response to bacterial lipoprotein	0.00912	CD14/CD36	2
0043312	neutrophil degranulation	0.00912	ITGB2/ITGAM	2
0019882	antigen processing and presentation	0.009222	FCER1G/CTSS/FCGR2B/CCL19	4
0019884	antigen processing and presentation of exogenous antigen	0.009323	FCER1G/CTSS/FCGR2B	3
1903557	positive regulation of tumor necrosis factor superfamily cytokine production	0.009337	TYROBP/CD14/CCL19/CD36	4
0071706	tumor necrosis factor superfamily cytokine production	0.009337	TYROBP/CD14/ACP5/CCL19/CD36	5

1903555	regulation of tumor necrosis factor superfamily cytokine production	0.009337	TYROBP/CD14/ACP5/CCL19/CD36	5
0006958	complement activation, classical pathway	0.009553	C1QA/C1QB/C1QC/IGKC	4
0002686	negative regulation of leukocyte migration	0.009581	SLAMF8/HMOX1/DPP4	3
0032611	interleukin-1 beta production	0.009974	TYROBP/ACP5/CCL19/CD36	4
0032651	regulation of interleukin-1 beta production	0.009974	TYROBP/ACP5/CCL19/CD36	4
0008038	neuron recognition	0.009974	CNTN4/NEXN/CXCR4	3
0043301	negative regulation of leukocyte degranulation	0.010059	FCGR2B/HMOX1	2
0060907	positive regulation of macrophage cytokine production	0.010059	LAPTM5/CD36	2
0001961	positive regulation of cytokine-mediated signaling pathway	0.010374	LAPTM5/CXCR4/MMP12	3
0044403	biological process involved in symbiotic interaction	0.010518	VAMP8/CCL8/CLEC5A/CXCR4/DPP4/TREM1	6
0031623	receptor internalization	0.010586	ITGB2/FCER1G/CD36/SELE	4
	cellular response to	0.010586	CCL8/CCL19/CHI3L1/CCL18	4

0071347	interleukin-1			
1903307	positive regulation of regulated secretory pathway	0.010713	ITGB2/VAMP8/ITGAM	3
0050864	regulation of B cell activation	0.01133	SLAMF8/TYROBP/LAPTM5/FCGR2B/IGKC	5
0002696	positive regulation of leukocyte activation	0.011791	ITGB2/TYROBP/VAMP8/ITGAM/IGKC/CCL19/DPP4	7
0017157	regulation of exocytosis	0.012227	ITGB2/VAMP8/ITGAM/FCGR2B/HMOX1	5
0006968	cellular defense response	0.012338	TYROBP/NCF2/CLEC5A	3
0002761	regulation of myeloid leukocyte differentiation	0.012737	CCR1/TYROBP/C1QC/CARTPT	4
0035641	locomotory exploration behavior	0.012794	ATP1A2/DPP4	2
0046348	amino sugar catabolic process	0.012794	NPL/CHI3L1	2
0002224	toll-like receptor signaling pathway	0.012896	PIK3AP1/CD14/CTSS/CD36	4
0032102	negative regulation of response to external stimulus	0.013155	SLAMF8/ACP5/FCGR2B/SPP1/DPP4/MMP12/CARTPT	7
0002440	production of molecular mediator of immune response	0.013203	LAPTM5/FCGR2B/HMOX1/IGKC/TREM1/CD36	6
0070372	regulation of ERK1 and ERK2 cascade	0.013279	CCR1/CCL8/CCL19/CHI3L1/CD36/CCL18	6

0042060	wound healing	0.013279	PLEK/FCER1G/HMOX1/MMRN1/CXCR4/CD36/MMP12	7
0060760	positive regulation of response to cytokine stimulus	0.01362	LAPTM5/CXCR4/MMP12	3
0001909	leukocyte mediated cytotoxicity	0.01362	TYROBP/ITGAM/FCGR2B/TREM1	4
0002468	dendritic cell antigen processing and presentation	0.014043	FCGR2B/CCL19	2
0006959	humoral immune response	0.014751	C1QA/C1QB/C1QC/FCGR2B/IGKC/TREM1	6
0032612	interleukin-1 production	0.014956	TYROBP/ACP5/CCL19/CD36	4
0032652	regulation of interleukin-1 production	0.014956	TYROBP/ACP5/CCL19/CD36	4
1901214	regulation of neuron death	0.014969	ITGB2/TYROBP/C1QA/ITGAM/FCGR2B/HMOX1	6
0006956	complement activation	0.015651	C1QA/C1QB/C1QC/IGKC	4
0071219	cellular response to molecule of bacterial origin	0.016326	CD14/CD180/LY86/FCGR2B/CD36	5
0032731	positive regulation of interleukin-1 beta production	0.016376	TYROBP/CCL19/CD36	3
0048002	antigen processing and presentation of peptide	0.016376	FCER1G/CTSS/FCGR2B	3

	antigen			
0007599	hemostasis	0.016376	PLEK/FCER1G/TPH1/MMRN1/CD36	5
0010934	macrophage cytokine production	0.016713	LAPTM5/CD36	2
0010935	regulation of macrophage cytokine production	0.016713	LAPTM5/CD36	2
0042053	regulation of dopamine metabolic process	0.016713	ITGB2/ITGAM	2
0042069	regulation of catecholamine metabolic process	0.016713	ITGB2/ITGAM	2
0070593	dendrite self-avoidance	0.016713	CNTN4/NEXN	2
0070371	ERK1 and ERK2 cascade	0.016713	CCR1/CCL8/CCL19/CHI3L1/CD36/CCL18	6
0002687	positive regulation of leukocyte migration	0.016916	CCR1/PLA2G7/CCL8/CCL19	4
0006940	regulation of smooth muscle contraction	0.016993	ATP1A2/CNN1/MYOCD	3
0045670	regulation of osteoclast differentiation	0.016993	CCR1/TYROBP/CARTPT	3
0048247	lymphocyte chemotaxis	0.016993	CCL8/CCL19/CCL18	3
0042113	B cell activation	0.017292	SLAMF8/TYROBP/LAPTM5/CD180/FCGR2B/IGKC	6
0015909	long-chain fatty acid transport	0.017581	FABP5/CD36/FABP4	3

1903037	regulation of leukocyte cell-cell adhesion	0.017633	ITGB2/LAPTM5/FCGR2B/CCL19/DPP4/SELE	6
0030889	negative regulation of B cell proliferation	0.017922	TYROBP/FCGR2B	2
0050766	positive regulation of phagocytosis	0.018086	FCER1G/FCGR2B/CD36	3
0050921	positive regulation of chemotaxis	0.018908	CCR1/PLA2G7/CXCR4/CCL19	4
0032496	response to lipopolysaccharide	0.019147	CD14/CD180/LY86/ACP5/CD36/SELE	6
0002523	leukocyte migration involved in inflammatory response	0.019406	SLAMF8/SELE	2
0032026	response to magnesium ion	0.019406	CD14/FBP1	2
0034763	negative regulation of transmembrane transport	0.019406	ATP1A2/CASQ2/FABP5/MMP9	4
0038061	NIK/NF-kappaB signaling	0.019406	CD14/LAPTM5/CCL19/CHI3L1	4
0046718	viral entry into host cell	0.01973	VAMP8/CLEC5A/CXCR4/DPP4	4
1901224	positive regulation of NIK/NF-kappaB signaling	0.01973	CD14/LAPTM5/CCL19	3
0002449	lymphocyte mediated immunity	0.020346	C1QA/C1QB/C1QC/FCER1G/FCGR2B/IGKC	6
0042742	defense response to bacterium	0.020346	SLAMF8/FCER1G/ACP5/IGKC/TREM1/CD36	6

0002577	regulation of antigen processing and presentation	0.020821	FCGR2B/CCL19	2
1903531	negative regulation of secretion by cell	0.020821	VAMP8/FCGR2B/HMOX1/CARTPT	4
0033627	cell adhesion mediated by integrin	0.021737	ITGB2/DPP4/CYP1B1	3
0071216	cellular response to biotic stimulus	0.022014	CD14/CD180/LY86/FCGR2B/CD36	5
0032732	positive regulation of interleukin-1 production	0.022187	TYROBP/CCL19/CD36	3
0002689	negative regulation of leukocyte chemotaxis	0.022187	SLAMF8/DPP4	2
0061081	positive regulation of myeloid leukocyte cytokine production involved in immune response	0.022187	LAPTM5/CD36	2
0090026	positive regulation of monocyte chemotaxis	0.022187	CCR1/PLA2G7	2
0044409	entry into host	0.022187	VAMP8/CLEC5A/CXCR4/DPP4	4
0070997	neuron death	0.022606	ITGB2/TYROBP/C1QA/ITGAM/FCGR2B/HMOX1	6
0048662	negative regulation of smooth muscle cell proliferation	0.023505	CNN1/MYOCD/HMOX1	3
	regulation of cardiac	0.023858	ATP1A2/CASQ2	2

0010881	muscle contraction by regulation of the release of sequestered calcium ion			
0032693	negative regulation of interleukin-10 production	0.023858	TYROBP/FCGR2B	2
0006809	nitric oxide biosynthetic process	0.02407	ACP5/CYP1B1/CD36	3
0007159	leukocyte cell-cell adhesion	0.02526	ITGB2/LAPTM5/FCGR2B/CCL19/DPP4/SELE	6
0035640	exploration behavior	0.025597	ATP1A2/DPP4	2
1903306	negative regulation of regulated secretory pathway	0.025597	FCGR2B/HMOX1	2
0050878	regulation of body fluid levels	0.02765	VAMP8/PLEK/FCER1G/TPH1/MMRN1/CD36	6
0046209	nitric oxide metabolic process	0.028162	ACP5/CYP1B1/CD36	3
0002700	regulation of production of molecular mediator of immune response	0.028344	LAPTM5/FCGR2B/HMOX1/CD36	4
0001910	regulation of leukocyte mediated cytotoxicity	0.028766	TYROBP/ITGAM/FCGR2B	3
2001057	reactive nitrogen species metabolic process	0.028766	ACP5/CYP1B1/CD36	3
0001894	tissue homeostasis	0.02906	ACP5/SNX10/IGKC/SPP1/CARTPT	5

1904996	positive regulation of leukocyte adhesion to vascular endothelial cell	0.029281	ITGB2/SELE	2
0002833	positive regulation of response to biotic stimulus	0.030203	TYROBP/CD180/LY86/MMP12	4
0006937	regulation of muscle contraction	0.030579	ATP1A2/CNN1/CASQ2/MYOCD	4
0031214	biomineral tissue development	0.030579	CCR1/SNX10/IBSP/SPP1	4
0098742	cell-cell adhesion via plasma-membrane adhesion molecules	0.030741	CNTN4/ITGB2/NEXN/ITGAM/SELE	5
0045672	positive regulation of osteoclast differentiation	0.030969	CCR1/TYROBP	2
0051048	negative regulation of secretion	0.031228	VAMP8/FCGR2B/HMOX1/CARTPT	4
0110148	biomineralization	0.031228	CCR1/SNX10/IBSP/SPP1	4
0015908	fatty acid transport	0.031228	FABP5/CD36/FABP4	3
0032413	negative regulation of ion transmembrane transporter activity	0.031228	ATP1A2/CASQ2/MMP9	3
0045921	positive regulation of exocytosis	0.031228	ITGB2/VAMP8/ITGAM	3
0002221	pattern recognition receptor signaling	0.031331	PIK3AP1/CD14/CTSS/CD36	4

	pathway			
1901136	carbohydrate derivative catabolic process	0.031331	NPL/PDE8B/CHI3L1/MMP12	4
0150104	transport across blood-brain barrier	0.031816	ATP1A2/FABP5/CD36	3
0031349	positive regulation of defense response	0.031816	TYROBP/VAMP8/PLA2G7/FABP4/MMP12	5
0010882	regulation of cardiac muscle contraction by calcium ion signaling	0.03189	ATP1A2/CASQ2	2
0006869	lipid transport	0.03189	FABP5/PLTP/SPP1/AQP9/CD36/FABP4	6
0010232	vascular transport	0.032234	ATP1A2/FABP5/CD36	3
0048771	tissue remodeling	0.032234	ACP5/SNX10/SPP1/CARTPT	4
0052126	movement in host environment	0.032234	VAMP8/CLEC5A/CXCR4/DPP4	4
1905475	regulation of protein localization to membrane	0.032234	ITGB2/VAMP8/ITGAM/MRAP2	4
1903532	positive regulation of secretion by cell	0.032833	ITGB2/VAMP8/ITGAM/SPP1/CARTPT	5
0071466	cellular response to xenobiotic stimulus	0.033279	SLAMF8/FBP1/CXCR4/CYP1B1	4
0038094	Fc-gamma receptor signaling pathway	0.033294	FCER1G/FCGR2B	2
0015850	organic hydroxy compound transport	0.033365	PLTP/SPP1/AQP9/CD36/CARTPT	5

0070664	negative regulation of leukocyte proliferation	0.033365	TYROBP/FCGR2B/CCL8	3
0001503	ossification	0.034507	CCR1/SNX10/IBSP/MMP9/CLEC5A/SPP1	6
0048660	regulation of smooth muscle cell proliferation	0.034612	CNN1/MYOCD/HMOX1/MMP9	4
0045766	positive regulation of angiogenesis	0.035017	HMOX1/CXCR4/CYP1B1/CHI3L1	4
1904018	positive regulation of vasculature development	0.035017	HMOX1/CXCR4/CYP1B1/CHI3L1	4
0002429	immune response-activating cell surface receptor signaling pathway	0.035858	TYROBP/LAPTM5/FCER1G/FCGR2B/IGKC	5
0002757	immune response-activating signal transduction	0.035858	TYROBP/LAPTM5/FCER1G/FCGR2B/IGKC	5
0002690	positive regulation of leukocyte chemotaxis	0.035858	CCR1/PLA2G7/CCL19	3
0048659	smooth muscle cell proliferation	0.035858	CNN1/MYOCD/HMOX1/MMP9	4
0010880	regulation of release of sequestered calcium ion into cytosol by sarcoplasmic reticulum	0.035858	ATP1A2/CASQ2	2
0034368	protein-lipid complex remodeling	0.035858	PLTP/PLA2G7	2
	plasma lipoprotein	0.035858	PLTP/PLA2G7	2

0034369	particle remodeling			
0043304	regulation of mast cell degranulation	0.035858	VAMP8/HMOX1	2
0050858	negative regulation of antigen receptor-mediated signaling pathway	0.035858	LAPTM5/FCGR2B	2
0090314	positive regulation of protein targeting to membrane	0.035858	ITGB2/ITGAM	2
0002532	production of molecular mediator involved in inflammatory response	0.036475	SLAMF8/VAMP8/CD36	3
0050764	regulation of phagocytosis	0.036475	FCER1G/FCGR2B/CD36	3
0002695	negative regulation of leukocyte activation	0.037266	TYROBP/LAPTM5/FCGR2B/HMOX1	4
0002718	regulation of cytokine production involved in immune response	0.037266	LAPTM5/HMOX1/CD36	3
0033006	regulation of mast cell activation involved in immune response	0.037266	VAMP8/HMOX1	2
0033238	regulation of cellular amine metabolic process	0.037266	ITGB2/ITGAM	2
0070528	protein kinase C signaling	0.037266	PLEK/FIBIN	2
0001906	cell killing	0.037298	TYROBP/ITGAM/FCGR2B/TREM1	4

0006816	calcium ion transport	0.037298	CCR1/ATP1A2/CASQ2/CCL8/CXCR4/CCL19	6
0050808	synapse organization	0.038534	C1QA/C1QB/ITGAM/C1QC/FCGR2B/LRRN1	6
0010039	response to iron ion	0.038534	C1QA/HMOX1	2
0010644	cell communication by electrical coupling	0.038534	ATP1A2/CASQ2	2
0034367	protein-containing complex remodeling	0.038534	PLTP/PLA2G7	2
0090022	regulation of neutrophil chemotaxis	0.038534	CCL19/DPP4	2
0015837	amine transport	0.038534	ATP1A2/AQP9/CARTPT	3
0031341	regulation of cell killing	0.038534	TYROBP/ITGAM/FCGR2B	3
0042100	B cell proliferation	0.038534	TYROBP/CD180/FCGR2B	3
0007162	negative regulation of cell adhesion	0.038534	LAPTM5/FCGR2B/ADAMDEC1/CYP1B1/MMP12	5
0015849	organic acid transport	0.038534	ATP1A2/FABP5/AQP9/CD36/FABP4	5
0002285	lymphocyte activation involved in immune response	0.039663	CD180/FCER1G/FCGR2B/CCL19	4
0050777	negative regulation of immune response	0.039663	SLAMF8/FCGR2B/HMOX1/MMP12	4
	mature B cell	0.039663	SLAMF8/FCGR2B	2

0002335	differentiation			
0002431	Fc receptor mediated stimulatory signaling pathway	0.039663	FCER1G/FCGR2B	2
0045920	negative regulation of exocytosis	0.039663	FCGR2B/HMOX1	2
0062014	negative regulation of small molecule metabolic process	0.040621	ACADL/PLEK/FBP1	3
1902106	negative regulation of leukocyte differentiation	0.040621	C1QC/FCGR2B/CARTPT	3
1904063	negative regulation of cation transmembrane transport	0.040621	ATP1A2/CASQ2/MMP9	3
0071248	cellular response to metal ion	0.040785	FBP1/HMOX1/MMP9/FABP4	4
0051047	positive regulation of secretion	0.040785	ITGB2/VAMP8/ITGAM/SPP1/CARTPT	5
0045785	positive regulation of cell adhesion	0.040785	ITGB2/IBSP/CCL19/DPP4/CD36/SELE	6
0001662	behavioral fear response	0.040785	ATP1A2/DPP4	2
0014808	release of sequestered calcium ion into cytosol by sarcoplasmic reticulum	0.040785	ATP1A2/CASQ2	2
0034383	low-density lipoprotein particle clearance	0.040785	HMOX1/CD36	2

0060249	anatomical structure homeostasis	0.042379	ACP5/SNX10/IGKC/SPP1/CARTPT	5
0002768	immune response-regulating cell surface receptor signaling pathway	0.042584	TYROBP/LAPTM5/FCER1G/FCGR2B/IGKC	5
0002209	behavioral defense response	0.042584	ATP1A2/DPP4	2
1903514	release of sequestered calcium ion into cytosol by endoplasmic reticulum	0.042584	ATP1A2/CASQ2	2
0019058	viral life cycle	0.043372	VAMP8/CCL8/CLEC5A/CXCR4/DPP4	5
1903707	negative regulation of hemopoiesis	0.043372	C1QC/FCGR2B/CARTPT	3
0051701	biological process involved in interaction with host	0.043611	VAMP8/CLEC5A/CXCR4/DPP4	4
0070661	leukocyte proliferation	0.043611	TYROBP/CD180/FCGR2B/CCL8/CCL19	5
0007229	integrin-mediated signaling pathway	0.043692	ITGB2/PLEK/ITGAM	3
0010876	lipid localization	0.043692	FABP5/PLTP/SPP1/AQP9/CD36/FABP4	6
0022407	regulation of cell-cell adhesion	0.043692	ITGB2/LAPTM5/FCGR2B/CCL19/DPP4/SELE	6
0090313	regulation of protein targeting to membrane	0.043692	ITGB2/ITGAM	2

0097009	energy homeostasis	0.043692	MRAP2/CD36	2
0016042	lipid catabolic process	0.043749	ACADL/PLCB4/PLA2G7/SPP1/CYP1B1	5
0071496	cellular response to external stimulus	0.043749	ATP1A2/MYOCD/HMOX1/MMP7/CARTPT	5
0003012	muscle system process	0.044959	ATP1A2/CNN1/CASQ2/MYOCD/HMOX1/CXCR4	6
0032410	negative regulation of transporter activity	0.045048	ATP1A2/CASQ2/MMP9	3
0034766	negative regulation of ion transmembrane transport	0.045048	ATP1A2/CASQ2/MMP9	3
0002698	negative regulation of immune effector process	0.045611	SLAMF8/FCGR2B/HMOX1	3
0006939	smooth muscle contraction	0.045611	ATP1A2/CNN1/MYOCD	3
0018958	phenol-containing compound metabolic process	0.045611	ITGB2/ITGAM/TPH1	3
1905954	positive regulation of lipid localization	0.045611	PLTP/SPP1/CD36	3
0071222	cellular response to lipopolysaccharide	0.045873	CD14/CD180/LY86/CD36	4
0045637	regulation of myeloid cell differentiation	0.04634	CCR1/TYROBP/C1QC/CARTPT	4
0050866	negative regulation of cell activation	0.04634	TYROBP/LAPTM5/FCGR2B/HMOX1	4
	fear response	0.046587	ATP1A2/DPP4	2

0042596				
0030100	regulation of endocytosis	0.04681	CD14/CCL19/CD36/SELE	4
0002526	acute inflammatory response	0.046885	CD163/FCGR2B/TREM1	3
1901222	regulation of NIK/NF-kappaB signaling	0.046885	CD14/LAPTM5/CCL19	3
0002701	negative regulation of production of molecular mediator of immune response	0.048246	FCGR2B/HMOX1	2
0042417	dopamine metabolic process	0.048246	ITGB2/ITGAM	2
0002286	T cell activation involved in immune response	0.048745	FCER1G/FCGR2B/CCL19	3
0051209	release of sequestered calcium ion into cytosol	0.049469	ATP1A2/CASQ2/CCL19	3
0071675	regulation of mononuclear cell migration	0.049469	CCR1/SLAMF8/PLA2G7	3
0009612	response to mechanical stimulus	0.049469	ATP1A2/CXCR4/CHI3L1/MMP7	4
0010543	regulation of platelet activation	0.049469	PLEK/FCER1G	2
0071276	cellular response to cadmium ion	0.049469	HMOX1/MMP9	2
1901020	negative regulation of calcium ion	0.049469	ATP1A2/CASQ2	2

	transmembrane transporter activity			
1904994	regulation of leukocyte adhesion to vascular endothelial cell	0.049469	ITGB2/SELE	2
0007596	blood coagulation	0.049528	PLEK/FCER1G/MMRN1/CD36	4
0051283	negative regulation of sequestering of calcium ion	0.049752	ATP1A2/CASQ2/CCL19	3
0045121	membrane raft	5.84E-06	ATP1A2/ITGB2/CD14/ITGAM/MS4A4A/CNTN1/HMOX1/DPP4/CD36/SELE/ITLN1	11
0098857	membrane microdomain	5.84E-06	ATP1A2/ITGB2/CD14/ITGAM/MS4A4A/CNTN1/HMOX1/DPP4/CD36/SELE/ITLN1	11
0044853	plasma membrane raft	1.77E-05	ATP1A2/ITGB2/ITGAM/MS4A4A/HMOX1/CD36/SELE	7
0009897	external side of plasma membrane	2.84E-05	CCR1/ITGB2/CD14/ITGAM/FCER1G/CD163/FCGR2B/IGKC/CXCR4/CD36/SELE	11
0030667	secretory granule membrane	9.04E-05	ITGB2/TYROBP/CD14/VAMP8/ITGAM/FCER1G/FABP5/CLEC5A/CD36	9
0070820	tertiary granule	9.04E-05	ITGB2/VAMP8/ITGAM/FCER1G/CTSS/MMP9/CLEC5A	7
0070821	tertiary granule membrane	0.000203	ITGB2/VAMP8/ITGAM/FCER1G/CLEC5A	5
0035579	specific granule membrane	0.000519	ITGB2/VAMP8/ITGAM/CLEC5A/CD36	5
0042581	specific granule	0.000632	ITGB2/VAMP8/ITGAM/CLEC5A/CHI3L1/CD36	6

0062023	collagen-containing extracellular matrix	0.000635	ANGPTL1/C1QA/C1QB/C1QC/CTSS/MMP9/MMRN1/ADAMDEC1/ATRNL1	9
0031225	anchored component of membrane	0.000725	CNTN4/CD52/CD14/CNTN1/CNTN3/ITLN1	6
0005901	caveola	0.003894	ATP1A2/HMOX1/CD36/SELE	4
0045335	phagocytic vesicle	0.021062	VAMP8/CTSS/NCF2/CD36	4
0030139	endocytic vesicle	0.021062	VAMP8/CTSS/NCF2/CD163/DPP4/CD36	6
0005581	collagen trimer	0.042521	C1QA/C1QB/C1QC	3
0034364	high-density lipoprotein particle	0.042521	PLTP/PLA2G7	2
0101002	ficolin-1-rich granule	0.049559	ITGB2/FCER1G/CTSS/MMP9	4
0008305	integrin complex	0.049559	ITGB2/ITGAM	2
0001540	amyloid-beta binding	0.003739	ITGB2/C1QA/ITGAM/FCGR2B/CD36	5
0005324	long-chain fatty acid transporter activity	0.003739	FABP5/CD36/FABP4	3
0030246	carbohydrate binding	0.005267	CNTN1/FBP1/CLEC5A/ATRNL1/CHI3L1/SELE/ITLN1	7
0008236	serine-type peptidase activity	0.005267	CTSS/CPVL/MMP9/DPP4/MMP12/MMP7	6
0017171	serine hydrolase activity	0.005267	CTSS/CPVL/MMP9/DPP4/MMP12/MMP7	6

0033218	amide binding	0.006854	ACADL/ITGB2/C1QA/CD14/ITGAM/PLTP/FCGR2B/CD36	8
0038024	cargo receptor activity	0.00701	ITGB2/ITGAM/CD163/CD36	4
0008081	phosphoric diester hydrolase activity	0.01066	CCR1/PLCB4/HMOX1/PDE8B	4
0004252	serine-type endopeptidase activity	0.012773	CTSS/MMP9/DPP4/MMP12/MMP7	5
0042578	phosphoric ester hydrolase activity	0.012773	CCR1/ATP1A2/PLCB4/ACP5/FBP1/HMOX1/PDE8B	7
0019864	IgG binding	0.012773	FCER1G/FCGR2B	2
0004620	phospholipase activity	0.012773	CCR1/PLCB4/HMOX1/PLA2G7	4
0048020	CCR chemokine receptor binding	0.013023	CCL8/CCL19/CCL18	3
0008009	chemokine activity	0.013023	CCL8/CCL19/CCL18	3
0004222	metalloendopeptidase activity	0.013023	MMP9/ADAMDEC1/MMP12/MMP7	4
0008035	high-density lipoprotein particle binding	0.013468	PLTP/CD36	2
0001846	opsonin binding	0.016973	ITGB2/ITGAM	2
0070492	oligosaccharide binding	0.017701	SELE/ITLN1	2
0016298	lipase activity	0.017701	CCR1/PLCB4/HMOX1/PLA2G7	4

0042277	peptide binding	0.017701	ITGB2/C1QA/CD14/ITGAM/FCGR2B/CD36	6
0030169	low-density lipoprotein particle binding	0.017701	PLTP/CD36	2
0005518	collagen binding	0.021072	CTSS/MMP9/MMP12	3
0005178	integrin binding	0.021072	ITGB2/ITGAM/IBSP/SPP1	4
0140375	immune receptor activity	0.021072	CCR1/FCER1G/FCGR2B/CXCR4	4
0042379	chemokine receptor binding	0.021841	CCL8/CCL19/CCL18	3
0001848	complement binding	0.021841	ITGB2/ITGAM	2
0005319	lipid transporter activity	0.021952	FABP5/PLTP/CD36/FABP4	4
0001618	virus receptor activity	0.021952	CLEC5A/CXCR4/DPP4	3
0140272	exogenous protein binding	0.021952	CLEC5A/CXCR4/DPP4	3
0016493	C-C chemokine receptor activity	0.021952	CCR1/CXCR4	2
0019865	immunoglobulin binding	0.021952	FCER1G/FCGR2B	2
0019957	C-C chemokine binding	0.023137	CCR1/CXCR4	2
0001637	G protein-coupled chemoattractant receptor	0.024089	CCR1/CXCR4	2

	activity			
0004435	phosphatidylinositol phospholipase C activity	0.024089	CCR1/PLCB4	2
0004950	chemokine receptor activity	0.024089	CCR1/CXCR4	2
0038187	pattern recognition receptor activity	0.024089	CD14/CD36	2
0004629	phospholipase C activity	0.027115	CCR1/PLCB4	2
0071813	lipoprotein particle binding	0.031392	PLTP/CD36	2
0071814	protein-lipid complex binding	0.031392	PLTP/CD36	2
0008237	metallopeptidase activity	0.03358	MMP9/ADAMDEC1/MMP12/MMP7	4
0019956	chemokine binding	0.033726	CCR1/CXCR4	2
0004497	monooxygenase activity	0.034294	HMOX1/TPH1/CYP1B1	3
0004175	endopeptidase activity	0.034294	CTSS/MMP9/ADAMDEC1/DPP4/MMP12/MMP7	6
0005504	fatty acid binding	0.043406	FABP5/FABP4	2
0016712	oxidoreductase activity, acting on paired donors, with incorporation or reduction of molecular oxygen, reduced flavin or	0.044556	HMOX1/CYP1B1	2

	flavoprotein as one donor, and incorporation of one atom of oxygen	

Supplementary Table 3 : GO analysis results.
