# Supplementary material for: Identification of core genes associated with the anti-atherosclerotic effects of Salvianolic acid B and immune cell infiltration characteristics using bioinformatics analysis
Source: BMC Complement Med Ther. 2022 Jul 16;22:190. doi: 10.1186/s12906-022-03670-6 (PMC9288713; doi:10.1186/s12906-022-03670-6)
Supplement: Supplementary file 5 — Additional file 5: Supplementary Table 5. GSEA results. [file 12906_2022_3670_MOESM5_ESM.pdf]

ID/ Description	Enrichment Score	qvalue	Core_enrichment	Rank
KEGG_AUTOIMMUNE_THYROID_DISEASE	0.836777803	1.37E-09	CD86/HLA-DMA/HLA-DMB/HLA-DRA/GZMB/HLA-DPB1/HLA-DPA1/CD80/CD28/HLA-DQA1/HLA-B/HLA-DQB1/HLA-F/HLA-DOB/HLA-C/HLA-A/HLA-E/HLA-DOA/HLA-G/PRF1/IL10/CTLA4/CD40LG/CD40	2041
KEGG_CHEMOKINE_SIGNALING_PATHWAY	0.61476166	1.37E-09	CCL19/CCR1/CCL18/CXCR4/CCL8/HCK/RAC2/CXCL16/CXCL10/CXCL8/LYN/CCL2/FGR/ARRB2/DOCK2/CCL4/VAV1/CCL21/VAV3/TIAM1/ELMO1/CCL5/CXCL2/PIK3CG/CCL13/ARRB1/PRKCB/CSK/ADCY7/PLCB2/CX3CR1/CCR2/PIK3R5/PRKCD/NCF1/CXCL1/ITK/ADCY3/WAS/JAK3/CCL7/ADCY4/CXCL13/PIK3CD/PTK2B/CCR5/PPBP/CCR7/CXCL11/STAT1/PRKX/GRB2/CCL20/NFKBIA/GNG2/CXCL12/PLCB1/GNGT2/CXCL9/PIK3R3/GNG10/STAT2/NRAS/GNG5/CCL23/CXCL3/GRK6/PIK3CB/SHC3/CXCL14/PAK1/PREX1/CXCL5/CCL22/CCL16	2054
KEGG_CYTOKINE_CYTOKINE_RECEPTOR_INTERACTION	0.661014607	1.37E-09	CCL19/CCR1/CCL18/CXCR4/CCL8/CSF1R/IL10RA/TNFSF13B/IL2RG/CXCL16/IL7R/CXCL10/CXCL8/CCL2/CSF2RB/TNFSF10/TNFRSF21/TNFRSF1B/CCL4/CCL21/CCL5/PLEKHO2/CXCL2/IL6/IL18/CCL13/FLT1/IL6R/TNFSF8/IL1B/CD27/CSF2RA/CX3CR1/IL7/CCR2/KDR/IL10RB/TNFRSF17/CSF3R/IFNAR2/TNFRSF14/CXCL1/IL12RB2/IL17RA/TNFRSF11A/VEGFA/IL2RA/VEGFC/TNFRSF9/TNFSF15/IL3RA/CCL7/IFNGR1/CXCL13/IL2RB/CCR5/CSF1/PPBP/IL18RAP/CCR7/IFNGR2/LEP/IL4R/CXCL11/RELT/CCL20/TNFRSF10A/ACVRL1/IL10/CXCL12/CXCL9/OSM/IL18R1/PDGFB/IL1R2/IL21R/IL12RB1/TNFRSF1A/CCL23/CXCL3/FLT3/TNFSF13/CXCL14/LIF/CD40LG/IL1R1/CXCL5/CD40/TNFSF18/CCL22/CCL16/INHBB/IFNLR1/PRLR/KIT/TNFRSF10B/XCR1/CCR6/LTBR/TNFSF14	2273
KEGG_GRAFT_VERSUS	0.873595	1.37E-09	CD86/HLA-DMA/HLA-DMB/IL6/HLA-DRA/GZMB/HLA-DPB1/HLA-	1156

_HOST_DISEASE			DPA1/CD80/CD28/HLA-DQA1/IL1B/HLA-B/HLA-DQB1/HLA-F/HLA-DOB/HLA-C/HLA-A/HLA-E/HLA-DOA/HLA-G/PRF1	
KEGG_HEMATOPOIETIC_CELL_LINEAGE	0.761669649	1.37E-09	CD36/ITGAM/CD14/CSF1R/MME/IL7R/ANPEP/CR1/CD37/CD4/IL6/HLA-DRA/ITGA4/CD38/ITGA6/CD33/IL6R/IL1B/CSF2RA/IL7/MS4A1/CSF3R/CD2/IL2RA/CD3D/IL3RA/FCGR1A/CD1C/TFRC/CSF1/CD22/IL4R/CD3G/CD3E/IL1R2/ITGA2/CD8A/CD5/FLT3/CD1E/IL1R1/ITGA5	1970
KEGG_LEISHMANIA_INFECTION	0.775134831	1.37E-09	ITGAM/ITGB2/NCF2/HLA-DMA/HLA-DMB/PTPN6/CR1/NCF4/TLR2/FCGR2A/MAPK13/HLA-DRA/ITGA4/HLA-DPB1/HLA-DPA1/C3/CYBA/HLA-DQA1/PRKCB/IL1B/HLA-DQB1/HLA-DOB/MYD88/NCF1/IFNGR1/FCGR1A/IFNGR2/STAT1/HLA-DOA/IRAK1/FOS/NFKBIA/IL10	1225
KEGG_LYSOSOME	0.754682353	1.37E-09	ACP5/CTSS/LAPTM5/LIPA/CTSC/ATP6V0D2/CTSB/CTSH/CTSL/LGMN/NPC2/MAN2B1/GLA/SLC11A1/CTSD/CTSK/ACP2/TPP1/PLA2G15/GM2A/TCIRG1/FUCA1/CTSZ/MCOLN1/ATP6V0B/AP1B1/NEU1/CTSA/GALC/HEXB/NAGA/GUSB/LAMP3/ATP6V0D1/IGF2R/NAGPA/M6PR/ATP6V0C/GAA/GNS/GNPTAB/PSAP/NPC1/GALNS/ATP6AP1/PPT1/LAMP2/GNPTG/CTSG/HEXA/ASAH1/GGA2/CTSW/CTNS/GGA1/CLN3/SLC11A2/CTSV/ARSA/ARSB/MANBA/GGA3/ATP6V1H/AP1S2/DNASE2B/LAMP1/CD63	2269
KEGG_NATURAL_KILLER_CELL_MEDIATED_CYTOTOXICITY	0.676389232	1.37E-09	ITGB2/FCER1G/TYROBP/RAC2/CD48/PTPN6/HCST/TNFSF10/SYK/ICAM1/ITGAL/VAV1/VAV3/LCP2/GZMB/PIK3CG/PRKCB/HLA-B/PIK3R5/BID/IFNAR2/ICAM2/HLA-C/FYN/IFNGR1/PIK3CD/PTK2B/SH3BP2/HLA-A/HLA-E/IFNGR2/PLCG2/HLA-	1934

			G/GRB2/PRF1/TNFRSF10A/NFATC2/MICB/CD247/PIK3R3/LCK/SH2D1A/KLRD1/NRAS/PIK3CB/SHC3/PAK1	
KEGG_TOLL_LIKE_RECEPTOR_SIGNALING_PATHWAY	0.713340517	1.37E-09	SPP1/CD14/CD86/LY96/CXCL10/CXCL8/CCL4/TLR2/TLR1/TLR8/MAPK13/CCL5/IL6/CTSK/PIK3CG/CD80/TLR5/IL1B/PIK3R5/TLR7/TLR6/IFNAR2/MYD88/PIK3CD/IRF5/CXCL11/STAT1/IRAK1/FOS/NFKBIA/TRAF3/CXCL9/PIK3R3/CASP8/MAP3K8/IKBKE/PIK3CB/TLR4/CD40/LBP	2116
KEGG_INTESTINAL_IMMUNE_NETWORK_FOR_IGA_PRODUCTION	0.812701896	3.89E-09	CXCR4/CD86/TNFSF13B/HLA-DMA/HLA-DMB/IL6/HLA-DRA/ITGA4/ITGB7/HLA-DPB1/HLA-DPA1/CD80/CD28/HLA-DQA1/TNFRSF17/HLA-DQB1/HLA-DOB/MAP3K14/HLA-DOA/IL10/CXCL12/TNFSF13/CD40LG/CD40/ICOSLG/LTBR	2250
KEGG_B_CELL_RECEPTOR_SIGNALING_PATHWAY	0.726840573	4.47E-09	FCGR2B/PIK3AP1/RAC2/LYN/PTPN6/BTK/SYK/VAV1/VAV3/BLNK/LILRB3/IFITM1/RASGRP3/INPP5D/PIK3CG/CD72/DAPP1/PRKCB/CD79A/PIK3R5/NFKBIE/PIK3CD/CD22/PLCG2/GRB2/FOS/NFKBIA/NFATC2/PIK3R3/CARD11/NRAS	1581
KEGG_ALLOGRAFT_REJECTION	0.860764553	8.19E-09	CD86/HLA-DMA/HLA-DMB/HLA-DRA/GZMB/HLA-DPB1/HLA-DPA1/CD80/CD28/HLA-DQA1/HLA-B/HLA-DQB1/HLA-F/HLA-DOB/HLA-C/HLA-A/HLA-E/HLA-DOA/HLA-G/PRF1/IL10	1225
KEGG_NOD_LIKE_RECEPTOR_SIGNALING_PATHWAY	0.756482899	1.41E-08	CCL8/CXCL8/CCL2/TNFAIP3/CASP1/MAPK13/CCL5/CXCL2/IL6/IL18/NLRC4/PYCARD/CCL13/NOD2/IL1B/NLRP3/CARD6/NAIP/CARD8/CXCL1/BIRC3/CCL7/NFKBIA/CASP8/CASP5/RIPK2/PSTPIP1/NOD1	1737
KEGG_ANTIGEN_PROCESSING_AND_PRESENTATION	0.7264646	6.20E-08	CTSS/HLA-DMA/CTSB/HLA-DMB/CTSL/LGMN/CD4/HLA-DRA/CD74/HLA-DPB1/HLA-DPA1/HLA-DQA1/HLA-B/HLA-DQB1/HSPA6/HLA-F/HLA-DOB/TAP1/HLA-C/HLA-A/TAPBP/HLA-E/HLA-DOA/HLA-	1566

			G/TAP2/CIITA/CD8A/KLRD1/B2M	
KEGG_CELL_ADHESION_MOLECULES_CAMS	0.622601774	1.20E-07	ITGAM/ITGB2/SELE/CD86/HLA-DMA/HLA-DMB/PTPRC/SIGLEC1/ICAM1/ITGAL/CD4/PECAM1/CDH5/SELPLG/HLA-DRA/ITGA4/SELL/ITGA6/ITGB7/HLA-DPB1/HLA-DPA1/CD80/CD28/HLA-DQA1/HLA-B/HLA-DQB1/CLDN1/SELP/HLA-F/HLA-DOB/VCAM1/CD2/ICAM3/ICAM2/HLA-C/HLA-A/CD22/PDCD1LG2/SPN/HLA-E/HLA-DOA/HLA-G/CADM1/NLGN4X/CD226/SDC1/CDH3/CD8A/CADM3/CTLA4/CD40LG/ITGB8/CD274/CD40/ICOSLG/CD276/CLDN7/SDC3	2108
KEGG_COMPLEMENT_AND_COAGULATION_CASCADES	0.710992441	3.24E-07	C1QB/C1QC/C1QA/C3AR1/C5AR1/F13A1/C7/CFI/PLAUR/C2/CR1/VWF/PLAU/SERPINA1/C3/PLAT	436
KEGG_T_CELL_RECEPTOR_SIGNALING_PATHWAY	0.624514486	4.94E-07	PTPRC/PTPN6/VAV1/VAV3/LCP2/CD4/MAPK13/PIK3CG/CD28/PIK3R5/RASGRP1/ITK/NFKBIE/MAP3K14/CD3D/FYN/PIK3CD/CD3G/CD3E/GRB2/FOS/NFKBIA/IL10/NFATC2/CD247/PIK3R3/LCK/CARD11/CD8A/NRAS/MAP3K8/CTLA4/PIK3CB/GRAP2/PAK1/MALT1/CBLB/CD40LG/TEC/PRKCQ/ZAP70/CBL/VAV2/IKBKB/PPP3CA/NCK1/PAK2/TNF	2685
KEGG_TYPE_I_DIABETES_MELLITUS	0.788337426	8.86E-07	CD86/HLA-DMA/HLA-DMB/HLA-DRA/GZMB/HLA-DPB1/HLA-DPA1/CD80/CD28/HLA-DQA1/IL1B/HLA-B/HLA-DQB1/HLA-F/HLA-DOB/HLA-C/HLA-A/HLA-E/HLA-DOA/HLA-G/PRF1/ICA1	1366
KEGG_SYSTEMIC_LUPUS_ERYTHEMATOSUS	0.747043748	2.11E-06	FCGR2B/C1QB/C1QC/C1QA/CD86/C7/HLA-DMA/HLA-DMB/C2/FCGR2A/HLA-DRA/HLA-DPB1/HLA-DPA1/C3/CD80/CD28/HLA-DQA1/HLA-DQB1/HLA-	1255

			DOB/FCGR1A/HLA-DOA/IL10/CTSG	
KEGG_PRIMARY_IMMUNODEFICIENCY	0.776944233	2.11E-06	IL2RG/IL7R/PTPRC/BTK/CD4/BLNK/CD79A/TAP1/JAK3/CD3D/ADA/CD3E/TAP2/LCK/CIITA/CD8A/CD40LG/CD40/ZAP70	2103
KEGG_ASTHMA	0.831715243	6.09E-06	FCER1G/HLA-DMA/HLA-DMB/HLA-DRA/HLA-DPB1/HLA-DPA1/HLA-DQA1/HLA-DQB1/HLA-DOB/FCER1A/HLA-DOA/IL10/MS4A2/CD40LG/CD40	2041
KEGG_FC_EPSILON_RI_SIGNALING_PATHWAY	0.659746503	9.69E-06	FCER1G/RAC2/LYN/BTK/SYK/VAV1/VAV3/LCP2/MAPK13/INPP5D/PIK3CG/PRKCB/PIK3R5/PRKCD/FYN/PIK3CD/PLCG2/FCER1A/GRB2/PIK3R3/PLA2G2D/NRAS/PLA2G2A/MS4A2/PIK3CB	1805
KEGG_LEUKOCYTE_TRANSENDOTHELIAL_MIGRATION	0.594741276	1.33E-05	MMP9/ITGAM/ITGB2/NCF2/CXCR4/RAC2/NCF4/ICAM1/ITGAL/VAV1/VAV3/PECAM1/CDH5/MAPK13/CYBB/ITGA4/PIK3CG/CYBA/PRKCB/THY1/PIK3R5/RHOH/CLDN1/VCAM1/NCF1/ITK/TXK/RASSF5/PIK3CD/PTK2B/PLCG2/CXCL12/PIK3R3/RAPGEF4	1393
KEGG_JAK_STAT_SIGNALING_PATHWAY	0.547023844	1.70E-05	IL10RA/IL2RG/IL7R/PTPN6/CSF2RB/IL6/PIK3CG/IL6R/MYC/CSF2RA/SOCS3/IL7/PIK3R5/IL10RB/CSF3R/IFNAR2/IL12RB2/IL2RA/JAK3/IL3RA/IFNGR1/PIK3CD/IL2RB/IFNGR2/LEP/IL4R/STAT1/GRB2/STAT5A/IL10/STAT4/TYK2/OSM/PIK3R3/IL21R/STAT2/IL12RB1/PIM1/PIK3CB/LIF/CBLB/IFNLR1/PRLR/SPRED1/CCND3	2184
KEGG_CYTOSOLIC_DNA_SENSING_PATHWAY	0.699739733	3.88E-05	CXCL10/CASP1/CCL4/CCL5/IL6/IL18/PYCARD/IL1B/AIM2/RIPK3/NFKBIA/ZBP1/IKBKE/IRF7/IKBKB/ADAR/DDX58	2587
KEGG_PPAR_SIGNALING_PATHWAY	0.631500003	0.00015571	FABP4/FABP5/CD36/PLTP/LPL/ACSL5/NR1H3/MMP1/SCD/OLR1/GK/PPARG/CYP27A1/DBI/ACSL1/PCK2	893
KEGG_FC_GAMMA_R_MEDIATED_PHAGOCYT	0.576758402	0.000240921	FCGR2B/HCK/RAC2/PTPRC/LYN/SYK/DOCK2/VAV1/FCGR2A/VAV3/MARCKS/INPP5D/PIK3CG/PRKCB/ARPC1B/ASAP1/PIK3R5/PRKCD/SCIN/NCF1/WAS/FCGR1A	1138

OSIS			/PIK3CD/ARPC3/PLCG2/SPHK1/LIMK1	
KEGG_APOPTOSIS	0.57415794	0.000438487	CSF2RB/TNFSF10/PIK3CG/IL1B/PIK3R5/BID/MYD88/CASP10/MAP3K14/BIRC3/IL3RA/PIK3CD/IRAK1/PRKX/TNFRSF10A/NFKBIA/PIK3R3/CASP8/TNFRSF1A/PIK3CB/IL1R1/TNFRSF10B/CAPN1/BAX/AIFM1/IRAK2/IKBKB/PPP3CA/ATM/TNF/IRAK3/TP53/IL1RAP/FASLG/CASP3/BCL2L1/BIRC2/NFKB1/TRAF2/TRADD/FADD/CASP7	3586
KEGG_DILATED_CARDIOMYOPATHY	-0.536022554	0.000663339	LAMA2/ITGB5/CACNA2D1/ADCY2/ITGB1/ITGA3/CACNA1C/TPM1/SGCG/DAG1/TGFB3/ITGA9/ADCY9/CACNB2/TGFB2/ITGA11/SGCA/DES/DMD/ITGA8/SGCD/SGCB/TPM2/ITGA10/ITGA7/ADCY5/RYR2/ACTC1/PLN	1636
KEGG_ARRHYTHMOGENIC_RIGHT_VENTRICULAR_CARDIOMYOPATHY_ARVC	-0.561999419	0.000825759	EMD/CACNA2D2/ITGAV/ATP2A2/CACNB1/CTNNA1/ITGB3/TCF7L2/TCF7L1/LAMA2/ITGB5/CACNA2D1/ITGB1/ACTN1/CTNNA3/ITGA3/CACNA1C/SGCG/DAG1/ACTN4/ITGA9/CACNB2/CDH2/ITGA11/SGCA/DES/DMD/GJA1/ITGA8/SGCD/SGCB/ITGA10/ITGA7/ACTN2/RYR2	2680
KEGG_EPITHELIAL_CELL_SIGNALING_IN_HELICOBACTER_PYLORI_INFECTION	0.601967896	0.000923013	ATP6V0D2/CXCL8/LYN/MAPK13/CCL5/CSK/TCIRG1/ATP6V1A/ATP6V0B/ATP6V1B2/CXCL1/MAP3K14/ATP6V0D1/ATP6V0C/HBEGF/PLCG2/ATP6AP1/NFKBIA/ADAM10/ADAM17/NOD1/GIT1/PAK1/ATP6V1C1/ATP6V1H/ATP6V0E1/ATP6V0A1/IKBKB/CXCR2	2613
KEGG_VIRAL_MYOCARDITIS	0.595454525	0.001062133	ITGB2/CD86/RAC2/HLA-DMA/HLA-DMB/ICAM1/ITGAL/HLA-DRA/HLA-DPB1/HLA-DPA1/CD80/CD28/HLA-DQA1/HLA-B/HLA-DQB1/BID/HLA-F/HLA-DOB/CXADR/HLA-C/FYN/HLA-A/HLA-E/HLA-DOA/HLA-G/PRF1	1156
KEGG_HYPERTROPHIC_CARDIOMYOPATHY_H	-0.53284446	0.001425551	LAMA2/ITGB5/CACNA2D1/ITGB1/PRKAB2/ITGA3/CACNA1C/TPM1/SGCG/DAG1/TGFB3/ITGA9/CACNB2/TGFB2/ITGA11/PRKAA2/SGCA/DES/DMD/ITGA8/SGC	1636

CM			D/SGCB/TPM2/ITGA10/ITGA7/RYR2/ACTC1	
KEGG_PRION_DISEASES	0.686591231	0.001746227	C1QB/C1QC/C1QA/C7/CCL5/IL6/IL1B/FYN/PRKX	1125
KEGG_VASCULAR_SMOOTH_MUSCLE_CONTRACTION	-0.489254312	0.002114147	EDNRA/CALM1/RAF1/ADCY6/AVPR1A/ADRA1A/BRAF/MYL6/ROCK1/ARHGEF12/GNA11/ADCY2/PLA2G12A/ACTA2/CACNA1C/NPR2/ITPR1/PTGIR/ROCK2/CALD1/RAMP1/ADRA1B/ADCY9/PPP1CB/PPP1R12B/PPP1R12A/MYLK/MYL9/KCNMB1/ACTG2/AGTR1/KCNMA1/PRKG1/MYH11/PPP1R14A/ADCY5/NPR1/PLCB4	2468
KEGG_BUTANOATE_METABOLISM	-0.651734287	0.002361763	PDHA1/ACAT2/ALDH5A1/ACAT1/ALDH3A2/ALDH7A1/ALDH2/HMGCS1/BDH2/GAD1/HADH/OXCT1/ALDH1B1	1634
KEGG_PROPANOATE_METABOLISM	-0.663624827	0.003372576	ACACB/ACACA/MLYCD/ACSS2/PCCA/ACADM/ACAT2/SUCLG2/ALDH6A1/ACAT1/ALDH3A2/LDHB/ALDH7A1/ALDH2/SUCLA2/HIBCH/ACSS3/ALDH1B1	4078
KEGG_RIG_I_LIKE_RECEPTOR_SIGNALING_PATHWAY	0.557451546	0.007871335	CXCL10/CXCL8/MAPK13/RNF125/MAP3K1/CASP10/TRIM25/IFIH1/NFKBIA/TRAF3/CASP8/IKBKE/ISG15/IRF7/DHX58/TANK/IKBKB/DDX58/TNF/NFKB1/TRAF2/TRADD/FADD/MAPK12	3634
KEGG_VIBRIO_CHOLERAE_INFECTION	0.583510777	0.008566183	ATP6V0D2/PRKCB/TCIRG1/ATP6V1A/ATP6V0B/ATP6V1B2/ADCY3/ATP6V0D1/ATP6V0C/PLCG2/PRKX/ATP6AP1/PDIA4/KCNQ1/ATP6V1C1/ATP6V1H/ATP6V0E1	2128
KEGG_TIGHT_JUNCTION	-0.422063215	0.011422561	MPP5/TJP2/AMOTL1/MAGI3/MYH9/RRAS2/CASK/CTTN/ACTN1/CTNNA3/PPP2CB/TJP1/PARD3/RRAS/AKT3/MAGI2/ACTN4/MPDZ/JAM3/MYL9/GNAI1/PPP2R2B/MYH10/MYH11/ACTN2	1496
KEGG_GLYCOSPHINGOLIPID_BIOSYNTHESIS_GLOBO_SERIES	0.787829346	0.011542647	GLA/GBGT1/HEXB/NAGA/HEXA/ST3GAL1	1726

KEGG_BLADDER_CANCER	0.602596591	0.019973413	MMP9/CXCL8/MMP1/DAPK1/MYC/TYMP/VEGFA/VEGFC/THBS1/NRAS/RB1/RPS6KA5/CDKN1A/CDKN2A/RASSF1/CDH1/MDM2	2781
KEGG_CARDIAC_MUSCLE_CONTRACTION	-0.469239453	0.025671377	CACNB3/SLC9A6/TPM4/TNNC1/COX7A2/CACNA2D2/ATP2A2/CACNB1/CACNA2D1/COX7A1/CACNA1C/TPM1/CACNB2/TPM2/RYR2/ACTC1/ATP1A2	3383
KEGG_ASCORBATE_AND_ALDARATE_METABOLISM	-0.73428533	0.02588602	UGDH/ALDH3A2/ALDH7A1/ALDH2/ALDH1B1	1494
KEGG_SPHINGOLIPID_METABOLISM	0.623906923	0.031848699	GLA/UGCG/NEU1/GALC/SGPL1/ACER3/SPHK1/SPTLC2/ASAH1/ARSA/NEU3/SMPD3	2424
KEGG_ENDOCYTOSIS	0.411300485	0.031848699	CXCR4/CSF1R/IL2RG/ARRB2/DAB2/SMAP2/FLT1/ARRB1/HLA-B/ASAP1/KDR/HSPA6/SH3KBP1/HLA-F/IL2RA/PSD4/ARAP1/HLA-C/AP2S1/IL2RB/TFRC/CCR5/HLA-A/HLA-E/NEDD4L/HLA-G/RAB11FIP1/RAB5C/WWP1/AP2A2/PSD3/GIT2/GRK6/PLD2/GIT1/ARAP3/CBLB/EHD4/KIT/ADRB2/CBL/CLTA/DNM2/CXCR2/EPN1/DNM1/MDM2/PRKCZ/ACAP2	2844
KEGG_PORPHYRIN_AND_CHLOROPHYLL_METABOLISM	0.639668586	0.031848699	HMOX1/GUSB/BLVRB/FTH1/ALAS1/CP/COX10/BLVRA/HMOX2/COX15/UROS/ALAS2	4015
KEGG_TYROSINE_METABOLISM	-0.545110185	0.033568753	AOX1/LCMT2/ADH1C/TRMT11/ADH1A/TYRP1/ADH1B/HPD/MAOA/ADH5/MAOB/AOC3	1391

Supplementary Table 5 : GSEA results.
