# Supplementary material for: Identification of core genes associated with the anti-atherosclerotic effects of Salvianolic acid B and immune cell infiltration characteristics using bioinformatics analysis
Source: BMC Complement Med Ther. 2022 Jul 16;22:190. doi: 10.1186/s12906-022-03670-6 (PMC9288713; doi:10.1186/s12906-022-03670-6)
Supplement: Supplementary file 6 — Additional file 6: Supplementary Table 6. Turquoise module genes of WGCNA in GSE28829 and GSE43292. [file 12906_2022_3670_MOESM6_ESM.pdf]

Gene ID						
ACP5	ADAMDEC1	ADAP2	AIM1	ALOX5AP	AMICA1	ANPEP
AOAH	APOC1	APOE	AQP9	ARRB2	ATP1A2	ATP6V0D2
BAMBI	BCAT1	BCL2A1	BIN2	C15orf48	C1orf162	C1QA
C1QB	C1QC	C2	C3AR1	C5AR1	C7	CA2
CAPG	CCDC109B	CCL18	CCL2	CCL4	CCL5	CCL8
CCR1	CD14	CD163	CD180	CD36	CD48	CD52
CD69	CD84	CD86	CECR1	CEMIP	CFI	CHI3L1
CHIT1	CLEC2B	CLEC5A	CPM	CPVL	CR1	CSF1R
CSF2RB	CSPG4	CTSC	CTSH	CTSS	CXCL16	CXCL2
CXCR4	CXorf21	CYP1B1	CYTIP	CYYR1	DCSTAMP	DNAJC5B
DPP4	EDNRB	ELTD1	EMCN	EPB41L3	EVI2A	EVI2B
F13A1	FABP5	FBP1	FCER1G	FCGR2B	FERMT3	FGR
FPR1	FPR3	GAS2L3	GBP5	GIMAP2	GIMAP4	GIMAP6
GIMAP7	GMFG	GPR116	GPR183	GPR34	GPR65	GREM1
GZMK	HCK	HCLS1	HK2	HLA-DMA	HLA-DMB	HLA-DQB1
HPGD	HPGDS	HS3ST2	IBSP	IER3	IFI27	IFI44
IFI44L	IGJ	IGKC	IL10RA	IL18	IL1B	IL1RN
IL2RG	IL7R	IQGAP2	IRF8	ITGAM	ITGAX	ITGB2
KMO	KYNU	LAIR1	LAPTM5	LCP1	LGR6	LILRB4
LIPA	LPL	LUM	LXN	LY86	LY96	LYN
LYZ	MARCO	MCTP1	MME	MMP12	MMP7	MMP8
MMP9	MNDA	MPEG1	MRC1	MREG	MS4A14	MS4A4A
MS4A6A	MSR1	MX2	MXRA5	NCF2	NCKAP1L	NPL
OLR1	P2RX7	PIK3AP1	PLA2G7	PLAUR	PLBD1	PLD5
PLEK	PLIN2	PLTP	PRDM1	PTPRC	RAC2	RARRES1
RGS1	RNASE1	RNASE6	RSAD2	S100A9	SAMSN1	SCD
SDS	SEL1L3	SERPINF1	SFRP2	SGK1	SKAP2	SLA
SLAMF7	SLAMF8	SLC11A1	SLC16A10	SLC16A6	SLC18B1	SLC1A3
SLC28A3	SLC2A3	SLC31A2	SLCO2B1	SNX10	SPP1	ST14
STEAP4	SULT1C2	TFEC	THEMIS2	TM4SF18	TMEM176A	TMEM71
TNC	TNFSF10	TNFSF13B	TREM1	TYROBP	UCP2	VAMP8
VNN1	VNN2	VSIG4				

Supplementary Table 5: Turquoise module genes of WGCNA in GSE28829 and GSE43292.
